# Development of a thermophilic coculture for corn fiber conversion to ethanol

**DOI:** 10.1038/s41467-020-15704-z

**Published:** 2020-04-22

**Authors:** Dhananjay Beri, William S. York, Lee R. Lynd, Maria J. Peña, Christopher D. Herring

**Affiliations:** 1 Thayer School of Engineering, Dartmouth College, Hanover, NH 03755 USA; 2 Centre for Bioenergy Innovation, Oak Ridge National Laboratory, Oak Ridge, TN 37830 USA; 3 Complex Carbohydrate Research Center, University of Georgia, Athens, GA 30602 USA; 4 Department of Biochemistry and Molecular Biology, University of Georgia, Athens, GA 30602 USA; 5 Enchi Corporation, Lebanon, NH 03766 USA; 6 Department of Biological Sciences, Dartmouth College, Hanover, NH 03755 USA

**Keywords:** Metabolic engineering, Genomics, Applied microbiology

## Abstract

The fiber in corn kernels, currently unutilized in the corn to ethanol process, represents an opportunity for introduction of cellulose conversion technology. We report here that *Clostridium thermocellum* can solubilize over 90% of the carbohydrate in autoclaved corn fiber, including its hemicellulose component glucuronoarabinoxylan (GAX). However, *Thermoanaerobacterium thermosaccharolyticum* or several other described hemicellulose-fermenting thermophilic bacteria can only partially utilize this GAX. We describe the isolation of a previously undescribed organism, *Herbinix spp*. strain LL1355, from a thermophilic microbiome that can consume 85% of the recalcitrant GAX. We sequence its genome, and based on structural analysis of the GAX, identify six enzymes that hydrolyze GAX linkages. Combinations of up to four enzymes are successfully expressed in *T. thermosaccharolyticum*. Supplementation with these enzymes allows *T. thermosaccharolyticum* to consume 78% of the GAX compared to 53% by the parent strain and increases ethanol yield from corn fiber by 24%.

## Abstract (web summary)

Corn fiber is a difficult feedstock to utilize due to its recalcitrant hemicellulose. Here, the authors characterize the recalcitrant structures, isolate a new bacterium to consume the hemicellulose, identify its enzymes, and show the benefit with increased conversion of corn fiber to ethanol.

## Introduction

Conversion of cellulosic feedstocks into biofuels is challenging due to their high recalcitrance, typically addressed with thermochemical pretreatment followed by large amounts of cellulase and hemicellulase enzymes to release soluble carbohydrates^1,2^. Recently, there has been interest in low-capital Generation 1.5 projects that produce ethanol from corn fiber at existing corn ethanol facilities^3,4^. Corn fiber makes up about 10% of the weight of corn kernels and consists of cellulose and hemicellulose from the aleurone and pericarp layers of the corn kernel. In current corn ethanol facilities, corn fiber ends up in the Distillers Dried Grains with Solubles (DDGS)^5^. If the fiber could be economically converted to ethanol, leveraging existing infrastructure, it would allow the facility to produce up to 13% more ethanol per bushel of corn while preserving and enriching the protein components as animal feed^5,6^. The cellulosic ethanol produced would generate additional revenue while serving as a transitional proving ground for advanced technology.

Cellulolytic bacteria and bacterial consortia have been identified that are mesophiles, moderate thermophiles and extreme thermophiles. *Clostridium thermocellum* is a moderate thermophile that has been extensively characterized and is one of the most effective organisms described to date at deconstructing cellulosic biomass^7,8^. In particular, several recent studies have found *C. thermocellum* to be 2- to 4-fold more effective than commercial fungal cellulase at solubilizing both woody and herbaceous lignocellulosic feedstocks^9,10^. Its thermophilic operating temperature may also be advantageous for industrial biomass conversion^11^. However, *C. thermocellum* is unable to ferment most of the carbohydrate present in hemicellulose, which represents a large portion of lignocellulosic biomass. As a potential coculture partner, *Thermoanaerobacterium saccharolyticum* can utilize xylan and other hemicellulose carbohydrates. It has also been engineered to produce ethanol at >90% of theoretical yield and up to 70 g/L titer^12^. However, its pH optimum is 6 while that of *C. thermocellum* is pH 7. A similar organism, *Thermoanaerobacterium thermosaccharolyticum*, is more compatible with *C. thermocellum* since it grows well at pH 7. It can also consume hemicellulose carbohydrates such as xylan^13,14^.

Corn fiber has high hemicellulose content and its major hemicellulosic polysaccharide is glucuronoarabinoxylan (GAX). GAX consists of a backbone of 1,4-linked β-xylose residues that are often substituted with arabinose side chains and to a lesser extent with glucuronic acid residues. The type and distribution of the GAX side chains varies between species, tissues, and developmental stage^15^. Corn fiber GAX has been shown to be particularly recalcitrant because of its high degree of substitution as well as the variety and complexity of its substituents^16,17^. These GAXs contain additional side chains with unusual residues such as l-galactose and α-xylose, which are not common components of plant cell wall xylans^18,19^. It is accepted that a large cocktail of enzymes acting synergistically is required to breakdown this complex polymer^16,17^. However, the fine structure of some of these side chains is not yet completely elucidated and this knowledge is essential to identify the hydrolytic enzymes necessary for their deconstruction.

In this study, we evaluate the performance of *C. thermocellum* with and without coculture partners in fermenting corn fiber. Utilization of GAX is important because of the need for high ethanol yield from available carbohydrate, but the diverse structures and linkages make this challenging for even specialized xylanolytic organisms. We present the isolation of a thermophile that can utilize most of the recalcitrant corn GAX and show that enzymatic activities identified from this isolate can be heterologously expressed in a thermophilic ethanologen host to enable improved cellulosic ethanol production from corn fiber.

## Results and Discussion

### *C. thermocellum* efficiently digests corn fiber

Corn fiber from a wet-milling process as well as destarched corn bran (DCB) from a dry mill, were both digested readily by *C. thermocellum* without added enzymes or pretreatment aside from autoclaving. We measured 90% solubilization within 3 days and 95% within 5 days of fermentation at 10 g/L solids. By comparison, commercial fungal cellulase (CTEC2) only solubilized 23%, while autoclaving alone released 10 ± 1% (Table 1). The poor performance with fungal cellulase could be due to inhibition of *T. reesei* cellulases in CTEC2 by phenols released by DCB hydrolysis^20^. Consistent with this possibility, better performance of fungal cellulase on wet-milled corn fiber (WMCF) has been shown, with 43 and 56% solubilization using 5 and 20 mg/g protein/solids, respectively^9^.

**Table 1:** Solubilization of corn fiber by various biocatalysts.

Biocatalyst	Corn fiber type	Solids loading (g/L)	% carbohydrate solubilization
None	DCB	10	10%^a^
CTEC2 (5 mg/g)	DCB	10	20 ± 2%
CTEC2 (20 mg/g)	DCB	10	23 ± 1%
*C. thermocellum*	DCB	10	95 ± 2%
*C. thermocellum*	WMCF	20	^b^91%
*C. thermocellum* + *T. saccharolyticum*	WMCF	10	96 ± 3%
*C. thermocellum* + *T. saccharolyticum*	WMCF	20	92 ± 2%

All the incubations were carried out for 5 days. ^a^Denotes *n* = 2. ^b^Denotes *n* = 1. For all other values, *n* = 3. Source data are provided as a Source Data file.

To ferment both cellulose and hemicellulose, a sequential coculture of *C. thermocellum* with *T. saccharolyticum* was performed (Table 1)*. T. saccharolyticum* was originally isolated for its ability to utilize xylan, so we expected the utilization of the corn fiber hemicellulose to be very high. Surprisingly, only 38 ± 3% of the non-glucose sugars (measured by liquid Quantitative Saccharification (QS)) that were solubilized by *C. thermocellum* were found to be consumed by *T. saccharolyticum* at both 10 and 20 g/L solids loading. The remaining sugars were left over in the broth. Reinoculating this broth afresh did not result in further growth. Comparing acid-hydrolyzed (liquid QS) to unhydrolyzed broth by HPLC indicated that only 10% of the carbohydrate was present as monosaccharides. Fermentations with a coculture of *C. thermocellum* and *T. thermosaccharolyticum* on corn fiber showed 45 ± 5% consumption of the non-glucose sugars.

### Structural characterization of GAX oligosaccharides

**Fig. 1:**
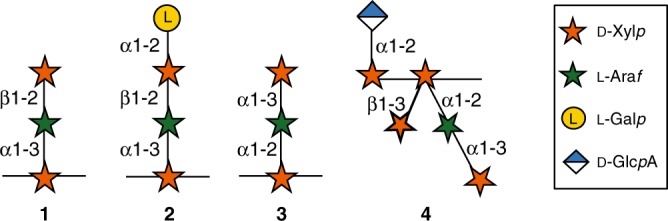
Structures of the four major side chains of the GAX oligosaccharides in the Coculture Broths. Orange stars: d-Xylopyranose, green stars: l-Arabinofuranose, yellow circles: l-Galactopyranose, blue and white diamonds: d-glucuronic acid. The structures were determined using NMR spectroscopy and MALDI-TOF MS spectrometry. Details of the structural characterization are shown in Supplementary Figs. 1 and 3 and Supplementary Tables 1, 2, and 3.

To identify the corn fiber carbohydrates solubilized but not utilized by *C. thermocellum* in monoculture and in coculture with *T. saccharolyticum* or *T. thermosaccharolyticum*, the fermentation broths were analyzed by NMR spectroscopy and MALDI-TOF mass spectrometry. These analyses showed that the main carbohydrates present in the broth after fermentation with *C. thermocellum* were arabinose and xylose monosaccharides, and xyloglucan and GAX oligosaccharides. Analysis of the Coculture Broths showed that the monosaccharides were almost completely consumed by *T. saccharolyticum* and *T. thermosaccharolyticum* but most of the GAX oligosaccharides remained unchanged with a degree of polymerization (DP) ranging from 4 to 20 (Supplementary Figs. 1 and 2). Xyloglucan oligosaccharides remained in the *TT*-Coculture Broth, meaning *T. thermosaccharolyticum* was unable to hydrolyze them. However, these oligosaccharides were absent in the *TS*-Coculture Broth suggesting *T. saccharolyticum’*s ability to consume them (Supplementary Fig. 2). For this reason, the *TS*-Coculture Broth was used to partially purify the GAX oligosaccharides by size-exclusion chromatography and to characterize their structure. Four main types of side chains (**1**–**4**) were identified in the GAX oligosaccharides in the broth (Fig. 1). The side chain **1** was identified as the disaccharide β-d-Xyl*p*-(1,2)-α-l-Ara*f*-(1,3) and represented 5% of the total carbohydrate in the *TS*-Coculture Broth (Fig. 1, Supplementary Table 1). This disaccharide is a common side chain in GAX from grasses^21^. The side chain **2** consisted of the disaccharide **1** with an α-l-Gal*p* residue attached at *O*-2 to the β-d-Xyl*p* and represented 13% of the total carbohydrate in the broth (Fig. 1, Supplementary Table 1). This side chain belongs to the series of side chains containing the unusual l-Gal*p* residue that are abundant in corn fiber but have also been found in other cereal GAXs^18,22,23^. The side chain **3** represented 13% of the total carbohydrate and contained α-d-Xyl*p*, another unusual sugar in plant cell wall xylans (Fig. 1, Supplementary Table 1). This disaccharide has been found previously in the pericarp of corn kernels^19^. The structure **4** was the most complex identified in the fermentation broth and represented 8% of the total carbohydrate (Fig. 1, Supplementary Table 2, Supplementary Fig. 3). This fragment contained two side chains, an α-d-Glc*p*A residue linked at *O*-2 to the backbone xylose at the non-reducing end and the following backbone xylose that was double substituted with a β-d-Xyl*p* residue at *O-*3 and the side chain **3** at *O*-2. The presence in corn fiber GAX of backbone xyloses double substituted with xylose and arabinose residues was proposed previously, but the oligosaccharide was never isolated or its structure characterized^16,24^. Interestingly, the oligosaccharides containing structure **4** were the only ones found in the Coculture Broths to contain uronic acids. Oligosaccharides carrying a single Glc*p*A or 4-*O*-Me-Glc*p*A side chains were identified in the Monoculture Broth and when GAX was extracted from corn fiber with alkali and digested with a xylanase (Supplementary Fig. 1). Their absence in the Coculture Broths indicated that enzymes in *T. saccharolyticum* and *T. thermosaccharolyticum* could hydrolyze simple uronic acid side chains and only the proximity to a large side chain in **4** prevented its cleavage.

### Bacterial utilization of GAX oligosaccharides

A survey of thermophilic organisms, including several described hemicellulose-utilizers, was conducted to find ones that were better than *T. saccharolyticum* and *T. thermosaccharolyticum* in utilizing the corn fiber GAX (Table 2). Bacteroides are gut bacteria that have been extensively studied for their ability to utilize complex glycans^25^ such as corn xylan^16^, and though it is not thermophilic, *Bacteroides cellulosilyticus* was included in this comparison. Only a few of the species could consume more than 60% of the total carbohydrate solubilized by *C. thermocellum*, consistent with the described recalcitrance of GAX (Table 2). Among these were *B. cellulosilyticus*, which utilized 85% of available carbohydrate, and *Caldanaerobius polysaccharolyticus* (AKA *Thermoanaerobacterium polysaccharolyticum*), a thermophile with described ability to breakdown and consume complex xylan^26–28^, which utilized 83%. A mixed thermophilic (55 °C) consortium maintained on switchgrass^29^ was also tested and found to consume 99% of the carbohydrate in the Monoculture Broth. This led to a quest to isolate organisms from the consortium with ability to utilize the recalcitrant corn fiber GAX.

**Table 2:** Utilization of carbohydrates in Monoculture Broth by various organisms.

Organism	% Utilization of carbohydrates in Monoculture Broth^a^
*Thermoanaerobacterium saccharolyticum*	58%
*Thermoanaerobacterium thermosaccharolyticum*	51%
*Caldanaerobius polysaccharolyticus*	83%
*Clostridium stercorarium*	61%
*Thermoanaerobacter ethanolicus*	28%
*Thermoanaerobacter mathranii*	49%
*Thermoanaerobacterium xylanolyticum*	22%
*Bacteroides cellulosilyticus*	85%
Thermophilic consortium^b^	99%

All calculations are based on at least four replicates. The CV is within ±10% for all values shown.

Source data are provided as a Source Data file.

^a^Monoculture Broth—Spent broth from a 20 g/L corn fiber fermentation of *C. thermocellum* which may have some residual *C. thermocellum* enzyme activity.

^b^Inoculum taken from semi-continuous anaerobic digester running at 55 °C on 30 g/L switchgrass.

### Isolation and sequencing of strain LL1355

In order to isolate an organism that is able to utilize the GAX oligosaccharides that *T. saccharolyticum* could not, the consortium was enriched and simplified on *TS*-Coculture Broth. Colonies were isolated on plates containing arabinose, cellobiose, and xylose. Sequencing of the 16S ribosomal RNA genes of 120 colonies revealed a variety of organisms, with the closest BLAST hit for the most abundant ones shown in Supplementary Table 3.

The isolated colonies were grown on *TS*-Coculture Broth to check for GAX utilization in liquid culture, but only two could grow. The pure cultures of these organisms, with closest BLAST matches to *Herbinix hemicellulosilytica* and *Ruminococcus champanellensis*, were named LL1355 and LL1354. They consumed 75% and 50% of the carbohydrates in the *TS*-Coculture Broth, respectively. Coculture of these two organisms did not give increased utilization. Also, LL1355 consumed 85% of the total carbohydrates in the Monoculture Broth.

Their genomes were sequenced by JGI, and the ORFs were analyzed using the dbCAN tool^30^ to get the list of all Carbohydrate-Active (CAZy) enzymes (Supplementary Table 4) The 16S ribosomal RNA gene sequence for LL1355 shows only 96.4% identity with *Herbinix hemicellulosylitica*, suggesting that we have isolated a species designated *Herbinix spp*. LL1355. BLAST results from housekeeping genes from the LL1355 genome show *Herbinix hemicellulosylitica* as the top hit, confirming the proposed genus assignment.

### Screening and characterization of enzymes from LL1355

Rogowski et al.^16^ characterized enzymes from *Bacteroides ovatus* involved in the breakdown of different xylans, including corn fiber GAX. In order to determine whether the same enzymes show activity on the GAX oligosaccharides released into solution by *C. thermocellum* fermentation, selected enzymes were tested for monosaccharide release on *TS*-Coculture Broth. Activity was observed for α-xylosidase-BoXyl31 (BACOVA_03422) and α-l-galactosidase-BoGal*p*95A. The arabinofuranosidase enzymes also showed activity when combined with these two (Supplementary Table 5).

Based on the elucidated structures of GAX oligosaccharides present in *TS*-Coculture Broth, the following LL1355 glycoside hydrolases were hypothesized to be important for degrading the recalcitrant linkages in GAX oligosaccharides: (i) α-d-xylosidase—GH31; (ii) α-l-galactosidase—GH95; (iii) α-l-arabinofuranosidase—GH43 and GH51; (iv) β-d-xylosidase—GH10 and GH120. In addition, α-d-galactosidase and α-glucuronidase enzymes were also indicated as small amounts of these residues were detected in the GAX side chains. In total, 27 ORF sequences from LL1355 were cloned and tested for activity, first by colorimetric assays measuring hydrolysis of sugar-nitrophenol complexes, then by checking for monosaccharide release from *TS*-Coculture Broth (Supplementary Table 6). All tests were done using cell-free extracts (CFE) of *E. coli* strains expressing LL1355 enzymes. Based on these tests, six enzymes, shown in Table 3, were selected for further study.

**Table 3:** Selected enzymes from LL1355.

Enzyme activity	Locus tag	Enzyme name	GH family
α-d-xylosidase	Ga0256695_1211	α-Xyl*p*_1211	31
α-l-galactosidase	Ga0256695_0687	α-l-Gal*p*_687	95
α-l-galactosidase	Ga0256695_0697	α-l-Gal*p*_697	95
β-d-xylosidase	Ga0256695_1710	β-Xyl*p*_1710	120
α-l-arabinofuranosidase	Ga0256695_0996	α-Ara*f*_996	43
α-l-arabinofuranosidase	Ga0256695_1120	α-Ara*f*_1120	43

The selected enzymes were purified using His-tag protein purification (Supplementary Fig. 4). The α-l-Gal*p*_697 proved difficult to purify. Since it had similar but lower activity than α-l-Gal*p*_687, it was not characterized further. Activity of the purified enzymes was determined using *p-*nitrophenyl glycosides (Supplementary Table 7). The specific activity of β-Xyl*p*_1710 is one of the highest reported for β-xylosidases^31–35^ while the measured activities on *p*NP α-l-arabinofuranoside were up to 100-fold lower than others^31,36^. There have been very few α-xylosidases described^37^. Most of them are active on xyloglucan oligosaccharides and/or on *p*NP α-d-xylopyranoside while only two have been shown to be active on corn xylan: BACOVA_03422 from *Bacteroides ovatus* and *Cj*Xyl31 from *Cellvibrio japonicus*^38^. The *Cj*Xyl31 has very low activity against corn xylan and prefers xyloglucan oligosaccharides. The specific activity of α-Xyl*p*_1211 on *p*NP α-d-xylopyranoside was higher than any previously reported values^39,40^.

GH95 enzymes are very rare and are generally α-l-fucosidases^41^. α-L-galactosidases are so uncommon as to not have an Enzyme Commission (EC) number or an entry in BRENDA. The only other reported α-l-galactosidase, *B. ovatus* α-Gal*p*95A, has been patented^16,42^. α-l-Gal*p*_687 was seen to have a higher activity than α-Gal*p*95A even at 37 °C. We compared the activity of the enzymes at 55 °C vs 37 °C and saw that in almost all cases more than 50% of the activity was retained at the lower temperature. Four out of the five enzymes from LL1355 were sensitive to oxygen though, with activities declining by up to 70% (Supplementary Table 7).

**Fig. 2:**
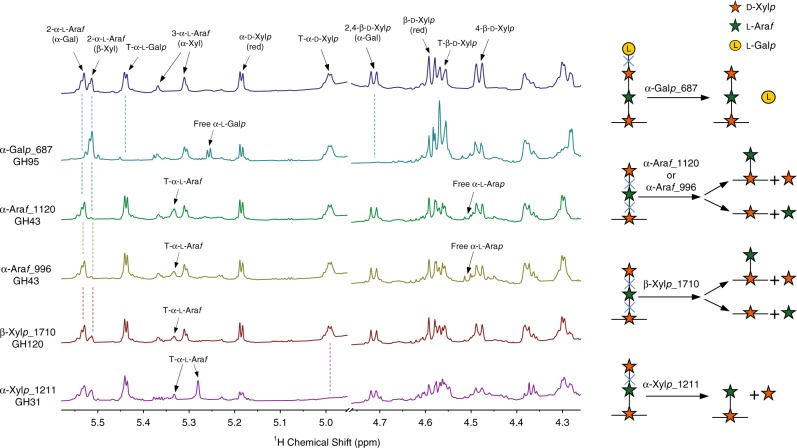
Activity of the purified enzymes against the Coculture Broth neutral oligosaccharides. 1D ^1^H NMR analysis of products generated by individually incubating the purified enzymes with a mixture of neutral oligosaccharides (DP 4-9) isolated from *TS*-Coculture Broth. Data correspond to 2 h incubation beyond which no further activity was detected. The signals labeled terminal (T) α-l-Ara*f* in the α-Ara*f*_1120, α-Ara*f*_996, and β-Xyl*p*_1710 spectra correspond to T-α-l-Ara*f* attached to *O*-3 to the backbone xylose. The T-α-l-Ara*f*-label in the α-Xyl*p*_1211 spectrum indicated the signal for T-α-l-Ara*f* attached to *O*-2. The xylose at the reducing end of the oligosaccharide are labeled red. A scheme of the enzymatic reaction for each enzyme is included on the right side of the figure.

To determine the capacity of the purified enzymes to hydrolyze the targeted linkages, the activities of the enzymes were individually tested against a mixture of GAX oligosaccharides purified from the *TS-*Coculture Broth. Due to the complexity of the oligosaccharide mixture in the broth, it was not possible to isolate individual oligosaccharides. The mixture consisted of neutral oligosaccharides (DP 4-9) containing the side chains **1**, **2**, and **3**. The substrate and the products of the reactions were analyzed by NMR and MALDI-TOF MS (Figs. 1 and 2, Supplementary Fig. 5, and Supplementary Table 1).

These analyses showed that α-l-Gal*p*_687 effectively removed the α-l-Gal*p* residues from the oligosaccharides containing the side chain **2**. The two α-arabinofuranosidases, α-Ara*f*_1120 and α-Ara*f*_996, completely removed the terminal β-d-Xyl*p* residue from the side chain **1**. The presence of signals in the NMR spectra for terminal α-l-Ara*f* and free Ara indicated that both enzymes also removed some of the α-l-Ara*f* residues from the side chain **1**, α-Ara*f*_996 being more effective than α-Ara*f*_1120. The enzyme β-Xyl*p*_1710 removed only some of the β-d-Xyl*p* residues from the side chain **1**. However, this is the only enzyme tested that was able to hydrolyze xylopentaose, a linear xylo-oligosaccharide (Supplementary Fig. 6).

**Fig. 3:**
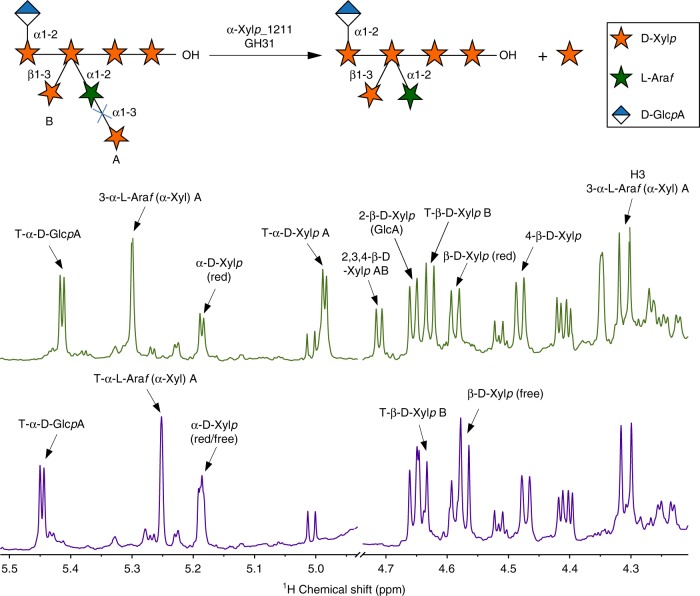
Activity of the α-xylosidase α-*Xylp*_1211 against the Coculture Broth acidic oligosaccharide. The GAX acidic oligosaccharide was isolated from the *TS*-Coculture Broth and incubated with the α-xylosidase α-Xyl*p*_1211 for 2 h. The oligosaccharide was analyzed by NMR before and after the incubation with the enzyme. The oligosaccharide contains a disaccharide α-d-Xyl*p* (1,3)-α-l-Ara*f*-(1,2)- (A) and a T-β-d-Xyl*p*-(1,3)-residue (B) that are attached to the double substituted 4-linked β-d-Xyl*p* in the backbone (AB). The disappearance of signal for terminal (T) α-d-Xyl*p* in the 1D ^1^H NMR spectrum of the oligosaccharide after the incubation, indicates that the enzyme effectively removed this residue from A. A diagram of the acidic oligosaccharide structure and the enzymatic reaction is included at the top of the figure.

The enzyme α-Xyl*p*_1211 completely removed α-d-Xyl*p* from the side chain **3**. The presence of two signals for this side chain in the NMR spectrum of the substrates indicated the existence of two different types of oligosaccharide carrying the side chain **3** in different locations. Both signals shifted downfield after the incubation with α-Xyl*p*_1211 indicating that the enzyme was active against the two types of structures. To determine if α-Xyl*p*_1211 and β-Xyl*p*_1710 were active against the complex structure **4**, acidic oligosaccharides containing this side chain were isolated and analyzed before and after the enzymatic treatment (Fig. 3; Supplementary Fig. 7, Supplementary Tables 2 and 8). These analyses showed that α-Xyl*p*_1211 was able to completely remove the α-d-Xyl*p* residues from structure **4**. However, β-Xyl*p*_1710 was not active against the side chain **4** even after the α-d-Xyl*p* residues were removed by α-Xyl*p*_1211.

This result was not surprising given that the β-d-Xyl*p* residue is surrounded by other sugar residues in this oligosaccharide. It is likely that the GlcA residue attached to the adjacent xylose limited the action of the β-xylosidase. Considering this, it is possible that side chain **3** is a product of *C. thermocellum* β-xylosidases, which could act against the double substituted xylose when it is not in the proximity of a GlcA residue.

Combinations of the selected enzymes were tested for monosaccharide release from *TT*-Coculture Broth (Supplementary Table 9), keeping in mind that the broth may have contained not just carbohydrate, but some residual enzyme activity as well. Structural analysis of the oligosaccharides after digestion confirmed that the targeted linkages had been hydrolyzed. We found a high degree of synergy between the enzymes, especially for the arabinofuranosidases, which have negligible effect on their own but considerable release of monosaccharides in conjunction with α-L-galactosidase and α-xylosidase. This can be understood structurally if the terminal glycosyl residue of the side chain needs to be removed before the other enzymes cleave the next residue. The five enzymes together deconstructed the oligosaccharides in the *TT*-Coculture Broth and released 42 ± 3% of the carbohydrate as monosaccharides (Supplementary Table 9). For comparison, the CFE from both LL1355 and *B. cellulosilyticus* released 45% of the carbohydrate as monosaccharides. Interestingly, the supernatant for all the tested organisms showed negligible activity (0–5%) (Supplementary Table 9), suggesting that the proteins are predominantly intracellular.

### Broth fermentations with enzyme supplementation

Combinations of enzymes were added to cultures of *T. thermosaccharolyticum* to measure the effect on utilization of carbohydrates in Monoculture Broth (Supplementary Table 10). Addition of all five enzymes allowed consumption of 78 ± 1% of the total carbohydrate while *T. thermosaccharolyticum* consumed only 53 ± 3% without enzyme addition. The most important enzyme for this purpose was α-Xyl*p*_1211, which when added alone resulted in 68 ± 2% of carbohydrate being utilized. Although α-L-Gal*p*_687 releases monosaccharides from *TT*-Coculture Broth, it did not greatly enhance carbohydrate utilization. This is due to the inability of *T. thermosaccharolyticum* to utilize the uncommon l-isomer of galactose that it releases. Addition of α-Xyl*p*_1211 and α-L-Gal*p_*687 along with any of the other three enzymes helped *T. thermosaccharolyticum* utilize almost the same amount of carbohydrate as addition of all enzymes (76% vs 78%, see Supplementary Table 10). This suggests that the α-Ara*f*_1120, α-Ara*f*_996, and β-Xyl*p_*1710 had similar activity on the carbohydrates in Monoculture Broth.

The effect of the addition of LL1355 CFE is also shown in Supplementary Tables 9 and 10. It is interesting to note that the performance of the five enzymes identified above and the LL1355 CFE was similar in catalyzing monosaccharide release (42 ± 3% vs 45 ± 4%), but the efficacy for increased carbohydrate utilization by *T. thermosaccharolyticum* was higher with the addition of LL1355 CFE (89 ± 1% vs 78 ± 1%). This may indicate the presence of additional enzymes in LL1355 CFE that did not show activity in our screen. An example of such an enzyme would be an endo-acting hydrolase, which might not release monosaccharides, but would nonetheless be important for efficient deconstruction. The existence of such enzymes is also suggested by considering *C. polysaccharolyticus* for which the endoxylanase Xyn10A has been reported to be important for xylan utilization^26^. It showed a high utilization (83%) of the *TT*-Coculture broth (Table 2) but its CFE and supernatant showed low activity in terms of monosaccharide release, at 17% and 0%, respectively (Supplementary Table 9). Taken together, results from both LL1355 and *C. polysaccharolyticus* suggest the potential to identify additional endo-acting hydrolases that may improve arabinoxylan utilization further.

### Expression of enzymes in *T. thermosaccharolyticum*

**Fig. 4:**
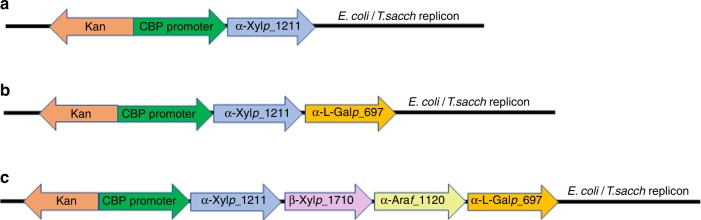
Expression constructs for glycosyl hydrolase expression in *T. thermosaccharolyticum*. **a** Construct for single enzyme expression, **b** construct for two genes which paired α-Xyl*p*_1211 with another enzyme, and **c** construct for expression of four genes together; three such constructs, as shown in Supplementary Table 11, were bulit. CBP promoter: strong promoter from *C. thermocellum* that drives the *cbp* (Clo1313_1954) gene; Kan: Kanamycin resistance marker; *E. coli/T. sacch* replicon: contains origins of replication for *E. coli* and *T. thermosaccharolyticum*/*T. saccharolyticum*.

Consolidated bioprocessing (CBP) entails production of required enzymes by the fermenting organism^11^. In order to enable CBP of corn fiber, we sought to express arabinoxylan degrading enzymes in *T. thermosaccharolyticum*, enhancing its ability to utilize corn fiber hemicellulose and thus improving product yield. The six selected enzymes in Table 3 were individually expressed in *T. thermosaccharolyticum* using a replicating plasmid construct shown in Fig. 4a. The activity of the cloned enzymes was confirmed in the CFEs of the resulting strains. The strain expressing α-Xyl*p*_1211 could utilize 32 ± 2% of the carbohydrates in the *TT*-Coculture Broth (Table 4) compared with 8 ± 5% by the parent strain. Subsequently, four 2-gene operons were constructed, all containing α-Xyl*p*_1211 plus another gene, then transformed into *T. thermosaccharolyticum* (Fig. 4b). All pairs of enzymes were determined to be active in CFEs.

**Table 4:** Utilization of carbohydrates in *TT or TS*-Coculture Broths by various *T. thermosaccharolticum* strains with heterologous expression of identified enzymes.

α-Xyl*p* _1211	α-l-Gal*p* _687	α-l-Gal*p* _697	α-Ara*f* _1120	α-Ara*f* _996	β-Xyl*p* _1710	% Carbohydrate utilization *TT*-Coculture Broth	Plasmid
Blank (Empty vector)						8 ± 5%	pLL1270
✓						32 ± 2%	pLL1271
✓			✓			34 ± 3%	pLL1272
✓				✓		37 ± 2%	pLL1274
✓	✓					38 ± 2%	pLL1273
✓		✓	✓	✓		44 ± 6%	pLL1277
✓		✓	✓		✓	48 ± 6%	pLL1275
✓		✓		✓	✓	49 ± 7%	pLL1276

Growth experiments were done on 2 different days with replicates *n* ≥ 4, with two different Coculture Broths from 20 g/L corn fiber fermentations: *TT*-Coculture Broth with 4.3 g/L or *TS*-Coculture Broth with 4.0 g/L total carbohydrates. Fresh media components were not added other than 5 g/L MOPS.

Cloning of 4-gene operons was attempted with α-Xyl*p*_1211, an α-L-galactosidase and two of the other three genes. Although α-l-Gal*p*_687 was initially included, operons containing that gene could not be successfully cloned, despite multiple attempts. Replacing it with α-l-Gal*p*_697 led to successful cloning. The three successfully expressed four-gene constructs are shown in Supplementary Table 11. Strains containing these constructs were transferred 8–10 times on *TT*-Coculture Broth to adapt them for better GAX utilization. The % carbohydrate utilization of all the developed strains is shown in Table 4. The best performing strain, expressing α-Xyl*p*_1211 + β-Xyl*p*_1710 + α-Ara*f* _996 + α-l-Gal*p*_697 (strain LL1703), was able to consume 49 ± 7% of the carbohydrate in *TT*-Coculture Broth after 10 days of incubation. Strains expressing α-Xyl*p_*1211 + α-l-Gal*p_*687 and any of the α-arabinofuranosidase enzymes utilized an average of 47 ± 1% of the carbohydrate in *TT*-Coculture Broth. For comparison, addition of LL1355 CFE resulted in a total utilization of 66 ± 1% on the same broth.

**Fig. 5:**
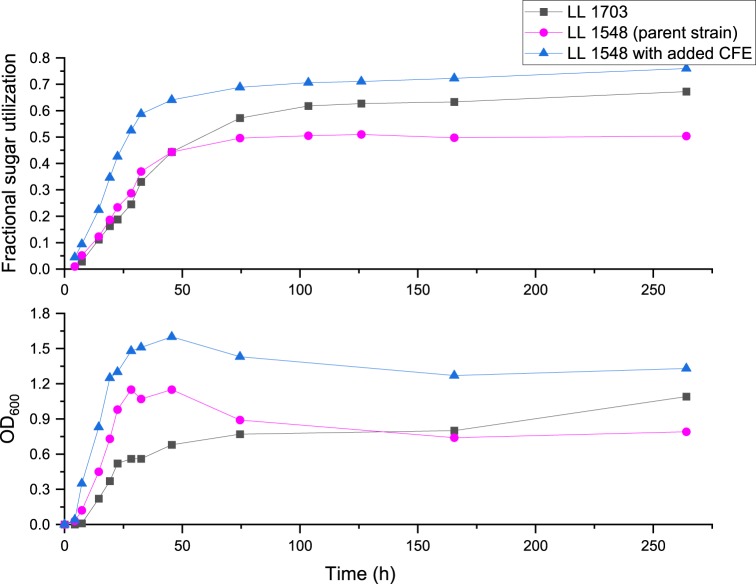
Growth of *T. thermosaccharolyticum* strain LL1703 on Monoculture Broth. The growth curves are representative of two replicates. The final % utilization (*n* = 6) was 65 ± 2% for the enzyme-expressing strain, 51 ± 1% for the parent stain, and 76 ± 2% for the strain supplemented with enzymes. Red circles—LL1548 (parent strain); black squares—LL1703 (LL1548 expressing four enzymes from LL1355); blue triangles—LL1548 with added LL1703 CFE. Source data are provided as a Source Data file.

In Monoculture Broth, the LL1703 strain utilized 67 ± 2% of the carbohydrates after 10 days, compared with 50 ± 2% by the parent strain. As shown in Fig. 5, LL1703 grew slower (lower OD_600_) than the parent strain, although both strains consumed the same amount of carbohydrates after 2 days. At this point in the growth curve, the parent strain stopped growing while LL1703 continued slowly consuming more carbohydrate even after 10–12 days, possibly due to enzyme release upon cells lysis. The difference in carbohydrate utilization between engineered strains expressing LL1355 enzymes and the parent strain is more distinct on *TT*-Coculture Broth, where the parent strain has no carbohydrate it can utilize, and lysis may happen earlier (Table 4). The *T. thermosaccharolyticum* parent strain culture supplemented with CFE from LL1703 showed faster growth on Monoculture Broth and utilized more carbohydrates than LL1703 itself (Fig. 5). The supernatant of LL1703 showed negligible activity in releasing monosaccharides from the *TT*-Coculture Broth (Supplementary Table 9). These results suggest that the heterologous enzymes were expressed intracellularly in LL1703 but extracellular expression of the selected enzymes is likely needed for optimal growth on GAX. It is notable that the selected enzymes from LL1355 did not have secretion signals and are likely expressed intracellularly. However, some CAZymes from LL1355 do have secretion signals, particularly endoxylanases. In line with these observations, the supernatant from a LL1355 culture did not have much activity in terms of monosaccharide release (Supplementary Table 9) but did increase the utilization of Monoculture broth carbohydrates by *T. thermosaccharolyticum* to 63% (Supplementary Table 10). The LL1355 genome contains a large number of oligosaccharide transporters that presumably allow the intake of the small GAX oligosaccharides resulting from the action of secreted endoxylanases. Similar to some human-gut *Bacteroides*, the preference for oligosaccharide uptake over secretion of processing enzymes may help the organism compete in the microbial communities that it has evolved to grow in^16^. *T. thermosaccharolyticum* may not have appropriate transporters for many of the oligosasaccharides in GAX and the benefit of the heterologous intracellular enzymes may not be fully realized.

### Corn fiber coculture fermentations with added enzymes/CFE

To study the solubilization of corn fiber fermentation with the addition of LL1355 or its CFE, pH-controlled bioreactor fermentations of 40 g/L fiber-enriched corn bran were conducted (Table 5). A coculture of *C. thermocellum* and *T. thermosaccharolyticum* solubilized 33% of the carbohydrate and consumed only 33% of the solubilized GAX. Addition of CFE from LL1355 increased GAX utilization to 64% and the solubilization to 46%, while coculture with LL1355 increased these values to 90% and 63%, respectively. These results indicate an association between increasing GAX utilization and increasing solubilization of the fiber. In another experiment, the five purified enzymes identified from LL1355 were added at a total loading of 1 mg/g (protein/corn fiber) to *C. thermocellum* and *T. thermosaccharolyticum* bottle fermentations on 8.8 g/L corn fiber. The ethanol titer increased to 2.4 ± 0.08 g/L compared with 1.93 ± 0.02 g/L for the control without enzyme addition (*n* = 3 for both).

**Table 5:** Fermentations on 40 g/L corn fiber using various biocatalysts.

Biocatalyst	% Carbohydrate solubilization	% GAX utilization
*C. thermocellum* + *T. thermosaccharolyticum*	33	33%
*C. thermocellum* + *T. thermosaccharolyticum* + LL1355 CFE	46	64%
*C. thermocellum* + LL1355	63	90%

Each reactor was run with two replicates. Source data are provided as a Source Data file.

Recent studies have shown inhibition of *C. thermocellum* by hemicellulose hydrolysis products, which establishes the need for a coculture partner proficient in hemicellulose utilization to assist in lignocellulose solubilization^43,44^. Engineering LL1355 for high ethanol yield could make it a useful coculture partner for *C. thermocellum* grown on corn fiber. We have shown improvement in solubilization with this coculture as compared with *C. thermocellum* alone or along with *T. thermosaccharolyticum*. Two other organisms, *Bacteroides cellulosilyticus* and *Caldanaerobius polysaccharolyticus*, showed high GAX utilization similar to LL1355, but *B. cellulosilyticus* is a mesophile while *C. polysaccharolyticus* grows poorly at 55 °C. Neither is a suitable coculture partner for *C. thermocellum*. However, enzymes from *C. polysaccharolyticus* in addition to endo-acting enzymes from LL1355 may warrant further study. The gap we observed between performance of heterologous enzyme expression and exogenous enzyme supplementation suggests further improvements (e.g., oligosaccharide transport or enzyme secretion) that may be required for full transfer of carbohydrate utilization from one organism to the other.

This study has demonstrated that *C. thermocellum* is much better than fungal cellulase at solubilizing the carbohydrate in corn fiber, achieving solubilization yield of >90%. This is substantially higher than its yields (<70%) on other lignocellulosic feedstocks such as corn stover or switchgrass^7,10^. We have also shown that various thermophiles described to be efficient xylanolytic organisms struggle to deconstruct and utilize corn GAX. To overcome this limitation, we exploited natural microbial diversity to isolate an organism *Herbinix spp*. strain LL1355 and used it as a source of enzymes for deconstruction of corn GAX. We have also demonstrated how a detailed structural characterization of the recalcitrant linkages in corn GAX helped in identifying enzymes for their processing, which could complement specific activities missing in our original host. Addition of just two enzymes, α-Xyl*p_*1211 and α-l-Gal*p_*687, increased utilization of complex corn GAX by *T. thermosacchrolyticum* from 53 ± 3% to 76 ± 1%. Addition of five enzymes resulted in a 24% improvement in ethanol titer from corn fiber by the thermophilic coculture of *C. thermocellum* and *T. thermosaccharolyticum*.

## Methods

### Fermentation conditions

Fermentations were carried out with *C. thermocellum* strain M1570^45^; the M1442 strain of *T. saccharolyticum*^46^ and LL1548, a high ethanol yielding derivative of *T. thermosaccharolyticum* strain LL1244, also known as ATCC 31960 and HG-8.

Wet-milled corn fiber was obtained from a Midwestern wet-milling facility and dry milled corn bran was obtained from Grain Millers, Inc. (Marion, IN). Destarched corn bran was prepared by treating 330 g of milled (0.5 mm) corn bran with 75 mL of α-amylase (Sigma-Aldrich A7595) and 25 mL of Spirizyme (Novozymes A/S, Bagsvaerd, Denmark) at 60 °C for 30 h followed by 5–6 water washes to get rid of all the solubilized starch. The bran was then dried in a 50 °C oven followed by milling to 0.2 mm.

Growth media were prepared as 10× concentrates and either passed through a 0.22 micron syringe filter (Corning Inc., Corning, NY) into a bioreactor or into autoclaved, nitrogen-flushed serum bottles. The media components were obtained from Sigma-Aldrich (St. Louis, MO, USA) or Fisher Scientific (Pittsburgh, PA). The composition of the 1X CC6 medium used for bioreactors was 5 g/L yeast extract, 2 g/L trisodium citrate*2H_2_O, 0.5 g/L K_2_HPO_4_, 0.5 g/L KH_2_PO_4_, 2 g/L MgSO_4_.7H_2_O, 0.1 g/L CaCl_2_, 0.1 g/L FeSO_4_*7H_2_O, 0.5 g/L L-cysteine and 1 g/L urea (for fermentations with *C. thermocellum* and *T. saccharolyticum*) or 1 g/L (NH_4_)_2_SO_4_ (for fermentations with *T. thermosaccharolyticum*). For bioreactors, a 50 mL (5%) inoculum of *C. thermocellum* was grown in a nitrogen-flushed 125 mL serum bottle with 10 g/L avicel PH-105 (FMC Corp., Philadelphia, PA) and CC6 medium supplemented with 5 g/L MOPS, pH set to 7.1 ± 0.1. *T. saccharolyticum* inoculum was prepared in the modified DSMZ 122 media^13^ adjusted to pH 6.5, and with 5 g/L cellobiose. *T. thermosaccharolyticum* inoculum was prepared in CTFUD^47^ with 5 g/L cellobiose, pH 6.7. For both these partner organisms, 50 mL inoculum was used in bioreactors.

Bioreactor fermentations were carried out in 3 L (1–2 L working volume) Biostat A-plus bioreactors (Sartorius Stedim, Bohemia, NY) with the temperature maintained at 55 °C using a resistive heat blanket and stirred at 200 rpm^48^. The pH was controlled at 6.95 with a Mettler-Toledo pH probe (Columbus, OH) by the addition of 4 N KOH. Base addition, pH and temperature were recorded by the MFSC/DA program included in the software accompanying the Sartorius A-plus bioreactor system^49,50^. The reactors were autoclaved for 1 h in a liquid cycle with only the feedstock and water. They were sparged with a mixture of 95% nitrogen and 5% carbon dioxide mixture (Airgas Northeast, White River Junction, VT) while cooling down to 55 °C. Media was then added into the reactor and pH was adjusted to 6.95 before inoculating. In case of sequential cocultures with *T. saccharolyticum*, fermentation was allowed to proceed with *C. thermocellum* for ~24 h before adjusting the pH to 6.25 with slow dropwise addition of 1 N HCl and then adding a 5% inoculum of *T. saccharolyticum*. Fermentations were carried out for 5 days unless otherwise noted.

### Culture broths

The broth resulting from fermentation was centrifuged to remove solids, then prefiltered through glass fiber filters without binder (MilliporeSigma, Burlington, MA). It was then filtered through a 0.45 µm Nylon Net Filter (HNWP04700, MilliporeSigma, Burlington, MA) and finally filtered through a 0.22 µm bottle-top filter (Corning Inc, Corning, NY) into a sterile bottle. The broth from a *C. thermocellum* fermentation is called ‘Monoculture Broth’, whereas the broths from a *C. thermocellum* plus *T. saccharolyticum* or *T. thermosaccharolyticum* fermentation are called ‘*TS*-Coculture Broth’ or ‘*TT*-Coculture Broth’, respectively.

### Carbohydrate composition analysis

The dry residual solids from a fermentation were analyzed using complete acid hydrolysis and HPLC (Quantitative Saccharification—QS)^49^ as follows. The residual solids were dried in a 55 °C oven till constant weight, which was noted. Approximately 0.1 g of the dried solids was then measured into a 50 mL round-bottom glass centrifuge tube (Kimble-chase 45212-50). 1.5 mL of conc. H_2_SO_4_ (for strong-acid hydrolysis) was added to the tube and a glass rod was put in for stirring. The tube was then incubated in a 30 °C water bath for 1 h with manual breaking of solid lumps at 15 min intervals. After the incubation, 42 ml of DI H_2_O was added immediately. The tubes were then capped tightly and autoclaved for 1 h (weak-acid hydrolysis) in a liquid cycle. This treatment converted the carbohydrates in the residual solids into monomer sugars that were then measured by HPLC as described below. Measured amounts of pure monosaccharides—glucose, arabinose, and xylose, were also taken through the same treatment and their final amount measured to normalize for degradation of carbohydrate. The composition of wet-mill corn fiber, expressed as monomer sugar equivalents, was 24% glucose, 35% xylose + galactose, and 13% arabinose. For the destarched corn bran (DCB), the composition was 22% glucose, 38.5% xylose + galactose, and 16% arabinose. For the fiber-enriched corn bran, the composition was 17.5% glucose, 23% xylose + galactose, and 12% arabinose. The total soluble carbohydrate in the liquid broth was analyzed by a similar acid hydrolysis method we refer to as liquid QS, which requires only the weak-acid hydrolysis step. Broth was centrifuged to remove solids and 35 µL of 72% H_2_SO_4_ was added to 1 mL of clear liquid supernatant, then put in a 4 mL shorty vial (DWK Life Sciences, Millville, NJ) and capped with a butyl rubber stopper. These vials were then autoclaved for 1 h on liquid cycle, along with acidified sugar standards to obtain a normalizing factor that can control for degradation. All the polysaccharides/oligosaccharides in the liquid were hydrolyzed and were measured on HPLC (Waters, Milford, MA) using xylose, arabinose, and glucose HPLC standards. The separation was performed with an Aminex HPX-87H column (Bio-Rad, Hercules, CA) at 60 °C, with RI (refractive index) detection and a 5-mM sulfuric acid solution eluent at a flow rate of 0.6 mL/min. The fermentation products were measured using the same column but with 25 µL of 10% H_2_SO_4_ added to 500 µL of sample to acidify^51^. All HPLC samples were passed through a 0.22 micron centrifuge tube spin filter (Costar Spin-X 8161, Cole-Parmer, Vernon Hills, IL) before loading on the column. Empower 2 software was used for instrument control and data analysis (Waters Corporation, Milford, MA, USA).

### Fungal cellulase incubation

0.5 g of destarched corn bran (0.2 mm) in 40 mL Milli-Q water was autoclaved for 40 min (liquid cycle) in a 125 mL serum bottle. CTEC2 (Novozymes) cellulase enzyme solution was prepared by 10× dilution in water and sterilized by filtering through a 0.22 µm syringe filter. The protein concentration was then measured by Bradford assay (Pierce™ Coomassie Plus (Bradford) Assay Kit—Thermo Fisher Scientific). The enzyme along with 5 mL of 10× concentrated sodium acetate buffer (pH 5), 75 µL penicillin G (2 g/L), and Milli-Q purified water was added to bring the total volume up to 50 mL. Final concentrations were as follows: sodium acetate: 50 mM; penicillin G: 3 ppm; CTEC2: 5 or 20 mg protein/g of corn bran. The incubation was done at 55 °C for 120 h at 180 rpm in an orbital shaker. The % solubilization was calculated by weighing residual solids and measuring their sugar composition by QS. It was confirmed by measuring the sugar amount in the released soluble carbohydrate by liquid QS.

### Growth of various organisms on Monoculture Broth

The growth conditions for various organisms tested on the Monoculture broth is given in Supplementary Method 1. A microbial consortium was obtained from Xiaoyu Liang in our lab who was maintaining a semi-continuous anaerobic digester at 55 °C on switchgrass at 30 g/L solids loading and 20 day residence time^29^.

The growth of these organisms on Monoculture Broth was tested in two ways: (1) the Monoculture Broth was supplemented with 10 g/L MOPS. Its final sugar composition measured by liquid QS was 3.4 g/L arabinose, 1.7 g/L glucose and 8.8 g/L xylose for a total of 13.9 g/L sugars; (2) 50% Monoculture Broth was supplemented with 0.5X CC6 medium and 10 g/L MOPS. Its final sugar composition measured by liquid QS was 1.8 g/L arabinose, 1 g/L glucose, and 4.8 g/L xylose for a total of 7.6 g/L sugars. The pH in both cases was adjusted using 1 N NaOH or 1 N HCl to the optimum value for each organism. The organisms were also grown on CTFUD (5 g/L cellobiose) under the same conditions to confirm complete cellobiose utilization. The strains were transferred on the Monoculture Broth two times before using a 2% inoculum for the final culture (1 mL volume) in disposable 15 mL Falcon screwcap centrifuge tubes. Cultures were incubated for 5 days in an anaerobic chamber (Coy Laboratory Products, Grass Lakes, MI, USA) at the optimum temperature for each organism. The % utilization in all cases was calculated by measuring carbohydrate content before and after growth by liquid QS.

### Isolation of organisms

To simplify the consortium obtained from Xiaoyu Liang, it was transferred 10 times on *TS*-Coculture Broth using a 10% inoculum. To simplify the consortium further, a dilution-to-extinction series was made, with the inoculum serially diluted 1/10 until the last few dilutions did not show any growth. These simplified consortia were then plated using the same media (CC6) as was used for the coculture and 5 g/L each of xylose, arabinose, and cellobiose.

Nearly 150 colonies were sent for 16S ribosomal RNA gene sequencing and results were obtained for about 120 colonies. Most colonies were streaked out to ensure purity. The pure cultures of a *Ruminococcus*-like and *Herbinix*-like isolate (likeness determined by 16S ribosomal RNA gene sequence matches) were added to the lab’s strain collection as LL1354 and LL1355, respectively. Their genomic DNA was sequenced by the Joint Genome Institute. The ORF sequences for both strains were analyzed with dbCAN, which is a web server and database for automated Carbohydrate-active enzyme annotation^30^.

### Analysis of corn fiber xylan-oligosaccharides

Wet-milled fiber was washed sequentially with aqueous 80% (v/v) EtOH and absolute EtOH. The residue was incubated with MeOH–CHCl_3_ (1:1, v/v) for a least 2 h and then the suspension was filtered using a nylon mesh (0.45 µm). The residue was washed with acetone and air dried to obtain the alcohol insoluble residue (AIR)^52^. The AIR was incubated with a glucoamylase and an α-amylase (Spirizyme® and Liquozyme®, respectively; Novozymes) to remove any remaining starch, and treated for 16 h at room temperature with 1 M KOH containing 1% (w/v) sodium borohydride. The 1 M KOH extract, which contains mainly xylan, was adjusted to pH 5 with glacial acetic acid, dialyzed against deionized water, and lyophilized. The xylan-enriched material was dissolved on 50 mM sodium phosphate buffer, pH 7.0 and incubated for 16 h at 37 °C with *Ct*Xyl5A, a recombinant xylanase from *C. thermocellum*^53^. The oligosaccharides enzymatically generated were analyzed by MALDI-TOF MS and NMR.

Aliquots of the fermentation broths of *C. thermocellum* monoculture and *C. thermocellum* and *T. saccharolyticum* coculture were desalted using P-10 columns (GE Healthcare, Chicago, USA) using the manufacturer’s instructions and analyzed by MALDI-TOF MS and NMR. Signals of the anomeric protons in the 2D NMR spectra of *TS*-Coculture Broth were assigned to their corresponding glycosyl residues of the oligosaccharides in the sample. To determine their abundance, the areas of the signals in the 1D spectrum were determined by integration or by deconvolution in the case of overlapping peaks.

### MALDI-TOF mass spectrometry

Positive ion MALDI-TOF mass spectra were recorded using an Applied Biosystems Voyager-DE biospectrometry workstation, and the data collection software used was Bruker Daltonik FlexControl version 3.0 (Bruker Corporation, https://www.bruker.com). Oligosaccharides samples (5 μL of a 1 mg/mL solution) were incubated with 1 µL of a suspension of Dowex-50 cation exchanger resin for 1 h. After centrifugation, 1 µL of the supernatant was mixed with an equal volume of matrix solution (20 mg/mL 2,5-dihydroxybenzoic acid in aqueous 50% MeOH) and dried on MALDI target plate. Typically, spectra from 200 laser shots were summed to generate a mass spectrum.

### NMR spectroscopy

NMR spectra were recorded with a Varian Inova NMR spectrometer with a VnmrJ version 4.2 Revision A software (Agilent Technologies) operating at 600 MHz using a 5 mm cold probe and a sample temperature of 25 °C. Lyophilized oligosaccharides were dissolved in D_2_O (0.3 mL, 99.9%; Cambridge Isotope Laboratories, Tewksbury, MA, USA) and placed in a 3 mm NMR tube. All two-dimensional spectra (gCOSY, TOCSY, NOESY, HSQCAD, and gHMBCAD) were recorded using standard Varian pulse programs. The 1D and (^1^H–^1^H) homonuclear 2D experiments were acquired using Presat for water suppression. The TOCSY and NOESY mixing times were 80 and 200 ms, respectively, and 256 FIDS consisting of 1024 points were recorded. For high resolution gCOSY spectra, 800 FIDs were collected. For the (^1^H–^13^C) heteronuclear 2D experiments the number of collected complex points was 1024 for ^1^H-dimension and 200 time-increments were recorded in ^13^C-dimension. Chemical shifts are given in ppm relative to internal acetone (*δ*^1^H 2.225 and *δ*^13^C 30.89). All the NMR spectra were processed using MestReNova software (Mestrelab Research S.L., Santiago de Compostela, Spain).

### Cloning of enzymes from LL1355

The 57 glycoside hydrolase enzymes found in the genome sequence of LL1355 were annotated with putative enzymatic activities based on their top hits by BLAST (NCBI-NIH). 27 different enzymes were selected from LL1355 and cloned into *E. coli* using the pEXP-5-NT/TOPO (Thermo Fisher Scientific) plasmid. The plasmids were then transformed into T7-Express competent *E. coli* cells (New England Biolabs, Ipswich, MA) to obtain strains which were subsequently used for protein expression. An empty pEXP-5-NT plasmid was also cloned to serve as a negative control.

### Protein expression

Protein expression with pEXP-5-NT clones was performed using slight modifications to the manufacturer’s protocol^54^. The strains were grown in a 50 mL volume in baffled flasks with either LB broth, Miller (Fisher Scientfic) or Terrific Broth, modified (Sigma-Aldrich) with 50 μg/mL carbenicillin added. The pellet was resuspended in 2 mL KH_2_PO_4_ buffer (67 mM, pH 7.2) and 1X BugBuster reagent (MilliporeSigma). The cells were lysed with Ready-Lyse Lysozyme and DNase I (New England Biolabs) was added to reduce viscosity. Finally, sonication was performed using a micro-tip with 18 cycles of 10 s at 50% amplitude with 20 s intervals. The lysate was then centrifuged to remove cell debris, incubated at 55 °C for 30 min to precipitate host cell proteins, then centrifuged again to remove these proteins. The supernatant was filtered through a 0.22 micron syringe filter to obtain the cell-free extract (CFE).

### Protein purification

Protein purification was performed using Econo-Column® Chromatography Columns, 2.5 × 10 cm (Bio-Rad, Hercules, CA) packed with Ni-Sepharose High Performance histidine-tagged protein purification resin (GE Lifesciences, Pittsburgh, PA). Details are included in the Supplementary Method 2.

### Nitrophenyl assays

The following para-nitrophenyl compounds were used according to the expected activity of the enzyme: α-d-xylopyranoside, α-l-arabinofuranoside, β-d-xylopyranoside, α-d-galactopyranoside (Megazyme or Sigma-aldrich); α-l-fucopyranoside, α-d-fucopyranoside (Carbosynth). The reaction mix was made by adding 5 mL 67 mM KH_2_PO_4_ buffer (pH 7.2), glutathione (0.2 mL of a freshly prepared 3 mM stock solution), and 0.5 mL of 10 mM solution of the requisite nitrophenyl compound. Into 270 μL of this reaction mix, 30 μL of the CFE or purified enzymes was added, mixed, and then incubated at 55 °C. To stop the reaction, 1200 μL of 100 mM Na_2_CO_3_ was added. Finally, the absorbance at 400 nm was recorded using a spectrophotometer and activity measured by subtracting absorbance of the blank solution. A unit was defined as the amount of enzyme (mg) required to release 1 µmol of *p*-nitrophenol in 1 min under the assay conditions.

### Monosaccharide release tests

Crude enzyme CFE or purified enzymes were added to *TT*-Coculture Broth in 1:9 ratio with the final enzyme concentration being 0.15 mg/mL. Incubation was done at 55 °C for 24 h. After incubation, the monosaccharides released by the added enzymes were determined by HPLC as above. The % monosaccharide release was calculated by comparing to total carbohydrate content measured by liquid QS.

### Determination of the activity of purified enzymes

The *TS*-Coculture Broth was fractionated by size-exclusion chromatography (SEC) using a Superdex-75 HR10-30 column eluted at 0.5 mL/min with water and refractive index detection. Under these conditions acidic oligosaccharides eluted from the matrix before neutral oligosaccharides of the same size^55^. The fractions containing oligosaccharides were collected manually and lyophilized. The acidic oligosaccharides were further purified from the neutral oligosaccharides using a LC-18 column (Supelclean; Sigma-Aldrich, USA) following the manufacturer’s instructions. The purified oligosaccharides were structurally characterized using MALDI-TOF MS and NMR. The fraction containing the acidic oligosaccharides and a second fraction containing a mixture of small neutral oligosaccharides (DP 4-10) were used as substrates to determine the hydrolytic activities of the purified enzymes. In addition, xylopentaose purchased from Megazyme was also used as substrate in the enzymatic assays.

The purified enzymes were dialyzed (6- to 8.5-kD molecular weight cutoff) against 50 mM KH_2_PO_4_ buffer, pH 7.2 to remove glycerol. The assays were performed in the same buffer containing 3 mg of the neutral oligosaccharide mixture and 6 μg of each purified protein. The reactions were carried out under N_2_ at 37 °C for 2 h. The products of the reactions were analyzed by MALDI-TOF MS and NMR.

The acidic oligosaccharides (5 mg) were incubated in the same buffer with 6 μg of α-Xyl*p*_1211 or β-Xyl*p*_1710 at 37 °C for 2 h under N_2_. After the reaction, the products were dialyzed against water and analyzed. The dialyzed product of the α-Xyl*p*_1211 reaction was incubated with 6 μg of β-Xyl*p*_1710 under the same conditions described above. The products of each reaction were analyzed by MALDI-TOF MS and NMR.

### Broth fermentations with enzyme supplementation

Enzyme-supplemented fermentations were performed with strain LL1548 in Monoculture Broth prepared from a 40 g/L corn fiber fermentation (12.9 g/L total carbohydrate) or from a 20 g/L corn fiber fermentation (6.5 g/L total carbohydrate). Supplementation was done with 0.15 mg protein/mL of crude *E. coli* cell extract or with 0.04 mg/mL of purified enzyme, respectively. A 2% inoculum was used from a LL1548 culture growing on Monoculture Broth. Enzyme-supplemented cultures were grown for 5 days in 1.5 mL screwcap tubes with O-ring seals (Westnet, Inc., Canton, MA). The remaining carbohydrate in the culture was measured by liquid QS.

### Corn fiber coculture fermentations with added enzymes/CFE

Bioreactor coculture fermentations of 40 g/L fiber-enriched corn bran were performed in Sartorius bioreactors as above. LL1355 inoculum was prepared in CTFUD media supplemented with 1.5 mg/L folate and 0.1 mg/L Vitamin B12 (Sigma-Aldrich). Bottle fermentations with 8.8 g/L corn fiber were carried out with a 10 mL culture volume, in nitrogen-flushed 30 mL serum bottles using CC6 media supplemented with 10 g/L MOPS. The *C. thermocellum* and *T. thermosaccharolyticum* inocula were prepared as for reactors, but 10% total inoculum volume was used. The bottles were incubated in a 55 °C shaker at 200 rpm for 5 days. An equimolar enzyme mix of all five purified LL1355 enzymes was used at a loading of 1 mg protein/g of substrate.

### Reporting summary

Further information on research design is available in the Nature Research Reporting Summary linked to this article.

## Supplementary information

Supplementary information

reporting summary

## Data Availability

A reporting summary for this Article is available as a Supplementary Information file. Data supporting the findings of this work are available within the paper and its Supplementary Information files. The datasets generated and analyzed during the current study are available from the corresponding author upon request. The genomic DNA for strains LL1354 and LL1355 was sequenced by the Joint Genome Institute (Bioprojects PRJNA371193 and PRJNA345749; nucleotide accession number NZ_RSDX01000001). Source data underlying Tables 1, 2, 4, and 5, as well as Fig. 5 are provided as a Source Data file.

## Source data

Source data
