# Supplementary material for: Development of a thermophilic coculture for corn fiber conversion to ethanol
Source: Nat Commun. 2020 Apr 22;11:1937. doi: 10.1038/s41467-020-15704-z (PMC7176698; doi:10.1038/s41467-020-15704-z)
Supplement: Supplementary file 1 — Supplementary information [file 41467_2020_15704_MOESM1_ESM.pdf]

Development of a thermophilic coculture for corn fiber conversion to ethanol

Beri *et al.*

Supplementary Method 1. Growth of various organisms on Monoculture Broth

Thermoanaerobacterium ethanolicus WT (JW200/ATCC 31550) was obtained from ATCC. It was grown in CTFUD media at pH 7 and 65°C. *Thermoanaerobacter mathranii* (DSM 11426) was obtained from DSMZ. It was grown in CTFUD media at pH 7.4 and 65°C. *Thermoanaerobacterium xylanolyticum* (DSM 7097) was obtained from DSMZ and was grown in CTFUD pH 6.2 and 55°C. *Clostridium stercorarium* (DSM 8532) was obtained from DSMZ and grown in CTFUD at pH 7 and 55°C. *Clostridium clariflavum* str. 4-2a was previously isolated from thermophilic compost enrichment cultures [1] and has been shown to be able to grow on monomeric xylose and solubilize xylan [2]. It was also grown on CTFUD at pH 7 and 55°C. *Bacteroides cellulosilyticus* (DSM 14838) was obtained from DSMZ , grown at 37°C anaerobically on CTFUD at pH 7.2 supplemented with 1 µg/mL vitamin K3 dissolved in ethanol, 0.005 µg/mL vitamin B12 in water and 1.2 µg/mL hematin dissolved in 0.2 mM L-histidine pH 8.0. 1000X stock solutions of these additions were used. *Caldanaerobius polysaccharolyticus* (DSM 13641) was obtained from Mascoma Corp (Strain No. M0034) under the old name *Thermoanaerobacterium polysaccharolyticum*. It was grown in CTFUD pH 7.0 at 65°C.

Supplementary Method 2. Protein purification

Large scale protein purification was performed using Econo-Column® Chromatography Columns, 2.5 × 10 cm (Bio-Rad, Hercules, CA) packed with Ni-Sepharose High Performance histidine-tagged protein purification resin (GE Lifesciences, Pittsburgh, PA). 500 mL of culture was grown in Terrific Broth, modified with added carbenicillin (50 µg/mL). The culture was induced overnight and then used to make 20 mL of cell free extract as described in the 'Methods' section. The buffer used to make the CFE was the same as the binding buffer for protein purification, supplemented with 1X cOmplete™, EDTA-free Protease Inhibitor Cocktail (Sigma Aldrich). The Binding buffer was 20 mM sodium phosphate, 500 mM

NaCl, 5 mM dithiothreitol (DTT, Government Scientific), pH 7.2. For all enzymes other than β-Xyl*p*_1710, 20 mM Imidazole (Sigma Aldrich) was also added to the binding buffer. β -Xyl*p*_1710 had a weaker binding to the resin, so no imidazole (Imi) was added. The Econo-column was put on a clamp and 2 mL of resin was added to it, the resin was washed with 10-15 mL of DI water to remove the ethanol (resin is stored in 20% ethanol) and then with 15 mL of Binding buffer to equilibrate the column. After equilibration, the filtered cell free extract was loaded on the column and protein was allowed to bind to the resin at room temperature for 1-2 h with continuous slow shaking. The column was put back and the bottom cap removed to collect the flow-through. The resin was then washed twice with 10 mL Wash buffer (Binding buffer with 50 mM Imi or 20 mM Imi in the case of β-Xyl*p*_1710). This was followed by a wash with 2 mL of buffer containing 100 mM Imi (50 mM- β-Xyl*p*_1710) and a final wash with 2 mL of buffer with 200 mM Imi (100 mM- β -Xyl*p*_1710). Finally, a series of 2 mL elutions was done with buffer containing 500 mM Imi until no protein could be detected in the elute by Bradford assay. Selected samples were run on a protein gel, and the pure samples were pooled together.

A concentration step of the purified proteins was carried out using 6 mL Corning Spin-X UF Concentrators (10K MWCO). The protein sample was diluted 2X with the storage buffer containing 100 mM KH_2PO_4, 20% glycerol, 5 mM DTT. Centrifugation was done at 7,000 xg for 15-20 min at a time. After the protein sample was concentrated, storage buffer containing 10% glycerol was added to the tube (repeated once) and centrifuged again, to exchange the buffer completely and remove the salt and imidazole. The purified proteins were frozen in small aliquots at -80 °C.

Supplementary Table 1. ^{1}H and ^{13}C NMR analysis of the neutral xylo-oligosaccharide in the *TS*-Coculture Broth before and after enzymatic degradation.

Side Chain /Residue	H1/C1	H2/C2	H3/C3	H4/C4	$H5_{a,b}$/C5	$H6_{a,b}$/C6
a						
T-β-D-Xyl*p*	4.57/103.6	3.28/73.7	3.45/76.4	3.63/69.9	3.98-3.33/65.9	
2-α-L-Ara*f*	5.51/107.2	4.28/90.0	4.11/76	4.19/84.4	3.84-3.73/61.7	
a after enzyme						
T-α-L-Ara*f*	5.34/109	4.18	3.97	4.19	3.82-3.72	
b						
T-α-L-Gal*p*	5.44/99.1	3.84/69.4	3.86/69.9	4.00/70.0	4.21/71.6	3.73/61.9
2-β-D-Xyl*p*	4.71/101.	3.53/77.5	3.71/77	3.62/70.1	3.97-3.36/65.9	
2-α-L-Ara*f*	5.53/107.	4.30/90.2	4.11/76	4.16/83.6	3.83-3.73/61.7	
c						
T-α-D-Xyl*p*	4.99/100.	3.56/72	3.7/73	3.64/70	3.73-3.64/62.6	
3-α-L-Ara*f*	5.31/109.4	4.39/80.5	3.91/85.5	4.30/84.1	3.83-3.76/62.0	
c after enzyme						
T-α-L-Ara*f*	5.28	4.17	3.95	4.15	3.8-3.7	

The terminal β-D-Xyl*p* residue in the side chain *a* (β-D-Xyl*p*-(1,2)-α-L-Ara*f*-(1,3)-) was removed for the action of the α-arabinosidase _1120 and 996, and the β-Xyl*p*_1710 to generate terminal (T) α-Ara*f*-attached to *O*3 to the xylose in the GAX backbone. The α-L-Gal*p*_687 removed L-Gal from the side chain *b* (α-L-Gal*p*-(1,2)-β-D-Xyl*p*-(1,2)-α-L-Ara*f*-(1,3)-) to generate free L-Gal and the side chain *a*. The α-xyl_1211 removed α-D-Xyl*p* from the side chain *c* (α-D-Xyl*p*-(1,3)-α-L-Ara*f*-(1,2)-) to generate the α-Ara*f*-1,2- side chain. Chemical shifts are reported in ppm relative to internal acetone, δ^{1}H 2.225 and δ^{13}C 30.89.

Supplementary Table 2. **Structural characterization by ^{1}H and ^{13}C NMR of the main acidic GAX oligosaccharide isolated from the *TS*-Coculture Broth.**

Residue	H1/C1	H2/C2	H3/C3	H4/C4	$H5_{a,b}$/C5
T-α-D-Glc*p*A	5.42/98.4	3.57/74.2	3.75/73.2	3.49/77.1	4.31/72.8
T-α-D-Xyl*p* (A)	4.99/100.5	3.55/72.2	3.74/73.9	3.64/70.2	3.55-3.73/62.8
3-α-L-Ara*f* (A)	5.30/108.8	4.35/80.1	3.92/85.3	4.27/83.7	3.83-3.76/62.0
T-β-D-Xyl*p* (B)	4.63/103.3	3.36/73.8	3.46/76.3	3.63/70.2	4.00-3.32/65.7
2-β-D-Xyl*p* (GlcA)	4.66/101.8	3.49/77	3.51/74.7	3.66/70.5	4.00-3.34/65.9
2,3,4-β-D-Xyl*p* (AB)	4.71/100.0	3.66/76.9	4.06/77.9	3.98/73.9	4.23-3.59/61.8
4-β-D-Xyl*p* (Int)	4.49/102.6	3.30/73.7	3.55/74.5	3.79/76.4	4.16-3.41/63.5
4-β-D-Xyl*p* (red)	4.59/97.3	3.25/74.7	3.55/74.6	3.79/76.5	4.05-3.37/63.5
4-α-D-Xyl*p* (red)	5.19/93.0	3.56/72	3.75/72	3.7/77	3.82-3.76/59.7

The glycosidic sequence of the main acidic oligosaccharide was determined to be [α-D-Glc*p*A-(1,2)]-β-D-Xyl*p*-(1,4)-[α-D-Xyl*p*-(1,3)-α-L-Araf-(1,2)]-[β-D-Xyl*p*-(1,3)]-β-D-Xyl*p*-(1,4)-β-D-Xyl*p*-. The disaccharide α-D-Xyl*p* (1,3)-α-L-Ara*f* -(1,2- (A) and T-β-D-Xyl*p* -(1,3)- residue (B) are attached to the double substituted 4-linked β-D-Xyl*p* in the backbone (AB). The T-α-D-Glc*p*A is attached at *O*2 of the β-D-Xyl*p* at the non-reducing end of the oligosaccharide. A chemical structure diagram of the acidic oligosaccharide is shown in Fig. 3 and also below. T stands for terminal; Int for internal Xyl; and red for reducing residues. Chemical shifts are reported in ppm relative to internal acetone, δ^{1}H 2.225 and δ^{13}C 30.89

Supplementary Table 3. Top BLAST hits of 16S ribosomal RNA gene sequence of organisms found in the simplified consortium transferred on *TS*-Coculture broth in order of abundance.

Top BLAST hits	% identity
Clostridium thermopalmarium	99%
Herbinix hemicellulosilytica/Mobilitalea sibirica	95-96%
Ruminococcus champanellensis/Anaerotruncus colihominis	93-94%
Defluvitalea saccharophila	96-97%
Clostridium thermosuccinogenes	99%
Clostridium cellulosi	98-99%
Tepidimicrobium ferriphilum	98%
Clostridium sphenoides	98-99%

Sequencing of the 16S ribosomal RNA genes of ~120 colonies revealed a variety of organisms, with the closest (highest %identity) BLAST hit for the most abundant ones shown here.

Supplementary Table 4. Glycosyl hydrolase enzymes in various organisms.

	Ruminococcus spp. LL1354	*Herbinix spp.* LL1355	*C.polysaccharolyticus* DSM13641	*T.thermosaccharolyticum* DSM571	*T. saccharolyticum* YS485	*C. thermocellum* DSM1313
GH1	2		2	2	4	2
GH2	6	5	4	1	1	1
GH3	5	2	4	2		2
GH4	2	1	2	3	2	
GH5	1	3	3	2	2	9
GH8	1	1				1
GH9	1					16
GH10	1	5	2	2	3	5
GH11						2
GH13	9		3	3	3	2
GH15				1	2	1
GH16						2
GH18	1	8	1	5	3	5
GH20			1			
GH23		1	2	2	2	2
GH25	2					
GH26	2			1	1	3
GH27	1	2				
GH28	1		1	1	2	
GH29	1	1	2	1		
GH30	2	1				2
GH31	4	3	3	2		
GH32	3			1		
GH35	1		1			
GH36	2	1	1	1	1	
GH38			2			
GH39	1		5	2		
GH42	1		3	1		
GH43	9	11	1	1	2	6
GH44	1					
GH48						2
GH51	1	4	3	1	2	1
GH52				1	1	
GH53	2		2			1
GH59					1	
GH65			2	1	2	
GH66	1				1	
GH67		1	1	1	1	
GH74						1
GH77	1					
GH78	3		1			
GH81						1
GH84					1	
GH88	1					
GH94	4	2	1	1		3
GH95	1	2	1			
GH105	2			1	1	
GH106	2		3			
GH109	2		8	3	4	
GH112	1					
GH115	1	1				
GH120		2		1	1	
GH124						1
GH126						1
GH127	1		2	1	1	
GH130	2		5	4	4	1
GH133						1
GH141						1
GH146	2	2	1			
GH154	1				1	
Total	88	59	73	49	49	75

GH: glycosyl hydrolase. The families considered important for cleavage of glycosyl bonds prevalent in arabinoxylan are highlighted in red. The data was obtained either from cazy.org for *T.*

thermosaccharolyticum DSM571, *T. saccharolyticum* YS485 and *C. thermocellum* DSM1313 or from dbCAN for *Ruminococcus spp*. LL1354, *Herbinix spp*. LL1355 and *C. polysaccharolyticus* DSM13641. An additional GH127 enzyme was added for *C. polysaccharolyticus* based on Wefers *et al.* [3]

Supplementary Table 5. % monosaccharide released from *TS*-Coculture broth by *Bacteroides ovatus* enzymes.

	arabinose	glucose	xylose
BoAbF 43B	0%	0%	0%
BoAbF 43D	0%	0%	0%
BoAbF 43A	0%	0%	0%
BoXyl 3A	0%	0%	0%
BoXyn 98A	0%	0%	0%
BoAbF 43E	0%	0%	0%
BoXyn 10A	0%	0%	0%
BoGal 95A	0%	0%	8%
BoXyl 31	3%	0%	5%
BoAbf 43C	0%	0%	0%
BoXyl 43A	0%	0%	0%
All enzymes	11%	0%	15%

n=1 for all enzymes/combinations tested. 10 µL of enzyme (1mg/mL) was added to 490 µL of *TS*-Coculture broth and incubated at 25^{0}C for 2 h. All enzymes were obtained from Nzytech (Lisbon, Portugal) (Bo- *Bacteroides ovatus*, Xyl- xylosidase, Gal- galactosidase, Xyn- Xylanase, AbF- arabinofuranosidase, number denotes the GH family). The *TS*-coculture broth contained 0.14 g/L arabinose, 0.08 g/L glucose and 0.22 g/L xylose + galactose as monomers initially. The total sugars present in the *TS*-coculture broth (measured by Liquid QS) were 1.04 g/L arabinose, 0.18 g/L glucose and 3.19 g/L xylose+galactose.

Supplementary Table 6. Screening of enzymes from LL1355 by measuring activity against *p*-Nitrophenyl compounds and on *TS*-Coculture broth.

Predicted activity (ORF #)	GH family	p-NP α-D-Xyl*p*	p-NP α-D-Gal*p*	p-NP α-L Ara*f*	p-NP β-D Xyl*p*	Monosaccharide release*
α-D-Xyl_1211	31	++	-	-	-	Yes
α-D-Gal_2181	36	-	+	-	-	-
α-L-Ara*f*_273	43	-	-	+	++	-
α-L-Ara*f*_1120	43	-	-	++	-	Yes
α-L-Ara*f*_2184	43	-	-	+	-	-
α-L-Ara*f*_1226	51	-	-	+++	-	-
α-L-Ara*f*_786	51	-	-	+	-	-
α-L-Ara*f*_996	43	-	-	+	++	Yes
β-D-Xyl*p*_1710	120	-	-	++	+++	Yes
α-L-Gal*p*_687	95	-	-	-	-	Yes
α-L-Gal*p*_697	95	-	-	-	-	Yes
16 other enzymes	various	-	-	-	-	-

The activity of the enzyme against each compound is shown in a range from No activity (-) to + < ++ < +++ denoting the strength of activity based on the intensity of color generated in the assay. The assay method is described in Methods. *p*-NP is para-nitrophenyl; α-D-Xyl*p*: α-D -D-xylopyranoside; α-L Ara*f* : α-L-arabinofuranoside; β-D Xyl*p*: β-D-xylopyranoside; α-D-Gal*p* : α-D-galactopyranoside

Supplementary Table 7. Specific activity of selected cloned enzymes on *p*-nitrophenyl substrates.

Enzyme	Specific activity (IU/mg)								
	*p*NP α-D-XylP		*p*NP α-L-AraF		*p*NP-β-D-XylP		*TT* Coculture Broth		O_2 inhibition
	55°C	37°C	55°C	37°C	55°C	37°C	55°C	37°C	
α-Xyl*p*_1211	5.1**	3.9					0.8	0.4	Yes
α-Ara*f*_1120			1.1**	0.80*					Yes
α-Ara*f*_996			0.4	0.2	2.2	1.0			Yes
β-Xyl*p*_1710			0.4	0.1	82**	41			Yes
α-L-Gal*p*_687							1.5	0.9	No

*p*NP α-D-XylP is *p*NP α-D-xylopyranoside; *p*NP α-L-AraF is *p*NP α-L-arabinofuranoside; *p*NP-β-D-XylP is *p*NP-β-D-xylopyranoside. For the *p*NP glycosides 1 Unit is defined as the amount of enzyme required to release 1 µmol of *p*-nitrophenol per minute. For *TT*-Coculture Broth 1 unit is defined as the amount of enzyme required to release 1 mg of monosaccharide (xylose, arabinose, galactose or glucose) per minute. All data based on at least n=3 except for where noted. The CV for all specific activity data on Nitrophenyl compounds (unless otherwise noted) is within ±15%. The CV for activity data for *TT*-Coculture Broth is ±30%. (*) denotes single replicate. (**) denotes ±30% CV.

Supplementary Table 8. ^{1}H and ^{13}C assignments of the acidic GAX oligosaccharide isolated from the *TS*-Coculture Broth after the incubation with α-Xyl*p*_1211 for 2 h.

Residue	H1/C1	H2/C2	H3/C3	H4/C4	$H5_{a,b}$/C5
T-α-D-Glc*p*A	5.46/98.3	3.58/74.2	3.75/73.2	3.48/77.1	4.31/72.8
T-α-L-Ara*f* (A)	5.25/108.5	4.317/82.0	3.93/77.5	4.09/85.0	3.83-3.72/62.0
T-β-D-Xyl*p* (B)	4.64/103.6	3.34/73.7	3.47/76.3	3.62/70.2	3.99-3.33/65.8
2-β-D-Xyl*p* (GlcA)	4.66/101.4	3.50/77.0	3.51/74.7	3.66/70.5	4.00-3.32/65.5
2,3,4-β-D-Xyl*p* (AB)	4.77/99.9	3.67/76.0	4.07/77.1	3.98/73.9	4.25-3.63/62.1
4-β-D-Xyl*p* (Int)	4.47/102.5	3.29/73.4	3.55/74.5	3.78/76.4	4.15-3.39/63.5
4-β-D-Xyl*p* (red)	4.59/97.4	3.25/74.9	3.55/74.6	3.78/77.0	4.06-3.38/63.7
4-α-D-Xyl*p* (red)	5.19/92.9	3.55/72	3.75/72	3.7/78	3.81-3.73/59.7

The products of the enzymatic reaction were analyzed by NMR and the glycosyl sequence of the oligosaccharides was determined as follows [α-D-Glc*p*A-(1,2)]-β-D-Xyl*p*-(1,4)-[α-L-Araf-(1,2)]-[β-D-Xyl*p*-(1,3)]-β-D-Xyl*p*-(1,4)-β-D-Xyl*p*. The terminal α-D-Xyl*p* attached at *O*3 to the Ara*f* (A) is removed by the α-Xyl*p*. A diagram of the acidic oligosaccharide structure and the enzymatic reaction is showed in the figure 3. T stands for terminal; Int for internal Xyl; and red for reducing residues. Chemical shifts are reported in ppm relative to internal acetone, δ^{1}H 2.225 and δ^{13}C 30.89

Supplementary Table 9. Monosaccharide release from *TT*-Coculture broth with the incubation of purified enzymes.

α-xyl 1211	α-L-gal 687	α-L-ArF 1120-2	α-L-Arf 996	β-xyl 1710	% Monosaccharide release		
					Ara	Xyl+Gal	Total
Blank					-	-	-
✓					30	18	18
	✓				1	10	7
				✓	3	4	3
✓		✓			32	18	18
✓				✓	33	24	22
✓	✓				31	26	23
	✓	✓			22	20	17
	✓		✓		16	18	14
	✓			✓	16	21	16
		✓	✓	✓	4	7	5
✓	✓	✓			52	36	33
✓	✓		✓		50	35	33
✓	✓			✓	48	41	36
✓	✓	✓	✓		56	37	35
✓	✓	✓		✓	57	45	40
✓	✓		✓	✓	53	44	39
✓	✓	✓	✓	✓	59	46	42
LL1355 CFE					61	51	45
Caldanaerobius polysaccharolyticus CFE					0	17	16
Bacteroides cellulosilyticus CFE					30	47	45
LL1355 Supernatant					7	6	5
Caldanaerobius polysaccharolyticus Supernatant					2	2	2
Bacteroides cellulosilyticus Supernatant					0	0	0
T. thermosaccharolyticum strain LL1703 CFE					33	26	26
T. thermosaccharolyticum strain LL1703 Supernatant					8	5	5

The data comes from two replicates done on the same day with one replicate done with a 3X diluted (with 67 mM KH_2PO_4 buffer, pH 7.2) *TT*-Coculture broth but with the same enzyme concentration of 1.5 mg/mL which was combine in a 1:9 ratio with the Coculture broth (Final enzyme concentration 0.15 mg/mL). The supernatant was concentrated by using a 10 kDa MWCO centrifugal filter. The data from the two replicates is quite similar because the incubation was done for a long time and the purpose was to determine the maximum amount of monosaccharides that could be released by the enzyme(s). The values for the two enzyme dosages agreed closely.

Supplementary Table 10. Increased utilization of carbohydrates from Monoculture broth with supplementation of enzymes to a *T. thermosaccharolyticum* culture.

α-Xyl*p* 1211	α-L-Gal*p* 687	α-L-Ara*f* 1120	α-L-Ara*f* 996	β-Xyl*p* 1710	% Carbohydrate Utilization-Monoculture Broth
Blank					53%
✓					68%
		✓			55%
			✓		58%
				✓	55%
✓		✓			71%
✓			✓		70%
✓				✓	70%
✓	✓				76%
✓	✓	✓			77%
✓	✓		✓		76%
✓	✓			✓	77%
✓	✓	✓	✓		78%
✓	✓	✓		✓	78%
✓	✓		✓	✓	78%
✓	✓	✓	✓	✓	78%
LL1355 CFE					89%
Caldanaerobius polysaccharolyticus CFE					70%
LL1355 Supernatant					64%
C. polysaccharolyticus Supernatant					63%

Check marks show which enzymes were included in each combination. The reported value is the average of experiments (n=1) done on two different days either with 0.15 mg/mL crude *E. coli* cell extract of enzyme added to 40 g/L corn fiber Monoculture broth (12.9 g/L total carbohydrate) or with 0.04 mg/mL of purified enzyme added to 20 g/L Monoculture broth (6.5 g/L total carbohydrate). The values obtained were very similar.

Supplementary Table 11. The three-gene constructs introduced into *T. thermosaccharolyticum*.

i.	α-Xyl*p*_1211 + β-Xyl*p*_1710 + α-Ara*f*_1120 + α-L-Gal*p*_697
ii.	α-Xyl*p*_1211 + β-Xyl*p*_1710 + α-Ara*f* _996 + α-L-Gal*p*_697
iii.	α-Xyl*p*_1211 + α-Ara*f*_1120 + α-Ara*f* _996 + α-L-Gal*p*_697

The order of genes is the same as in the operon cloned into the plasmid. Plasmid map is shown in Fig. 4.

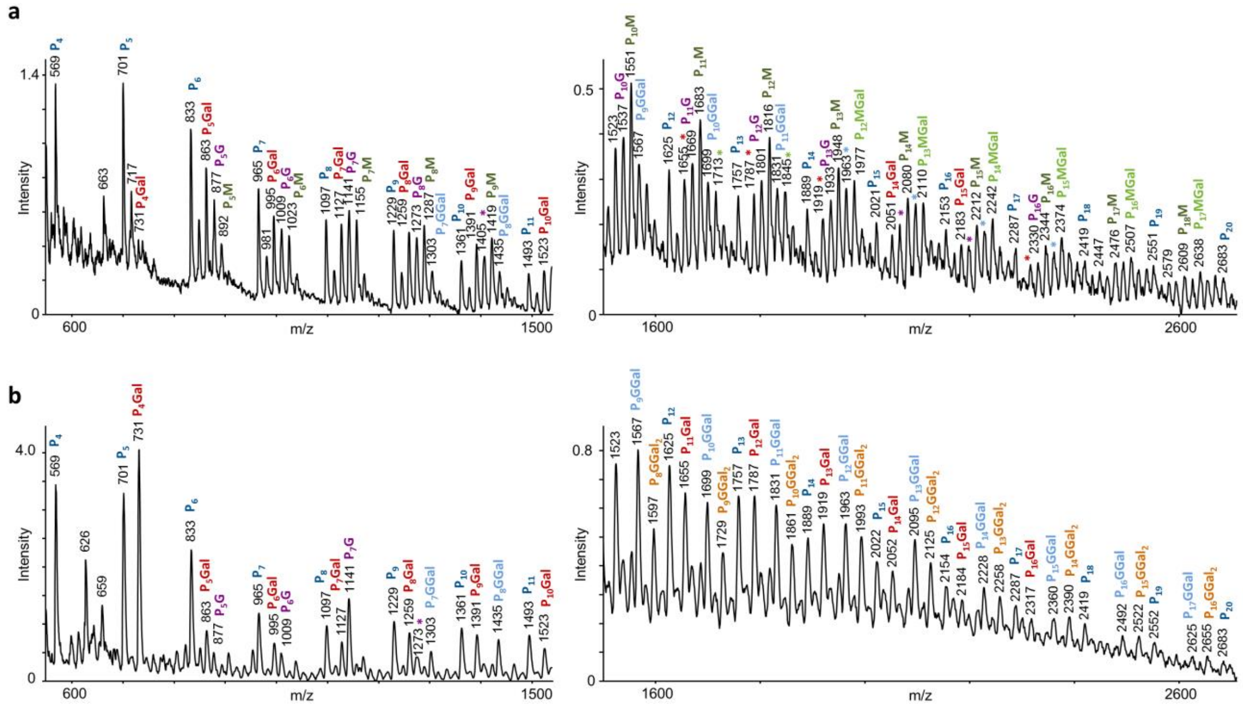

Supplementary Figure 1. MALDI-TOF mass spectra of the corn fiber GAX oligosaccharides. (a) Mass spectra of GAX oligosaccharides generated by first extracting GAX from corn fiber with 1M KOH and then treating them with *Ct*A5 xylanase (b) Mass spectra of GAX oligosaccharides in the *TS*-Coculture broth. This analysis showed that the degree of polymerization (DP) of the oligosaccharides in the samples ranges from 4 to 20. Two partial spectra of each sample are presented to show the less intense signals of the oligosaccharides of higher DP. Note the different scale of the spectra. P stands for pentose (arabinose or xylose), Gal for galactose, G for glucuronic acid and M for 4-*O*-methyl glucuronic acid. The oligosaccharides series are labeled with different colors. For reasons of space, some labels are substituted for starts of some color of the labels of the series.

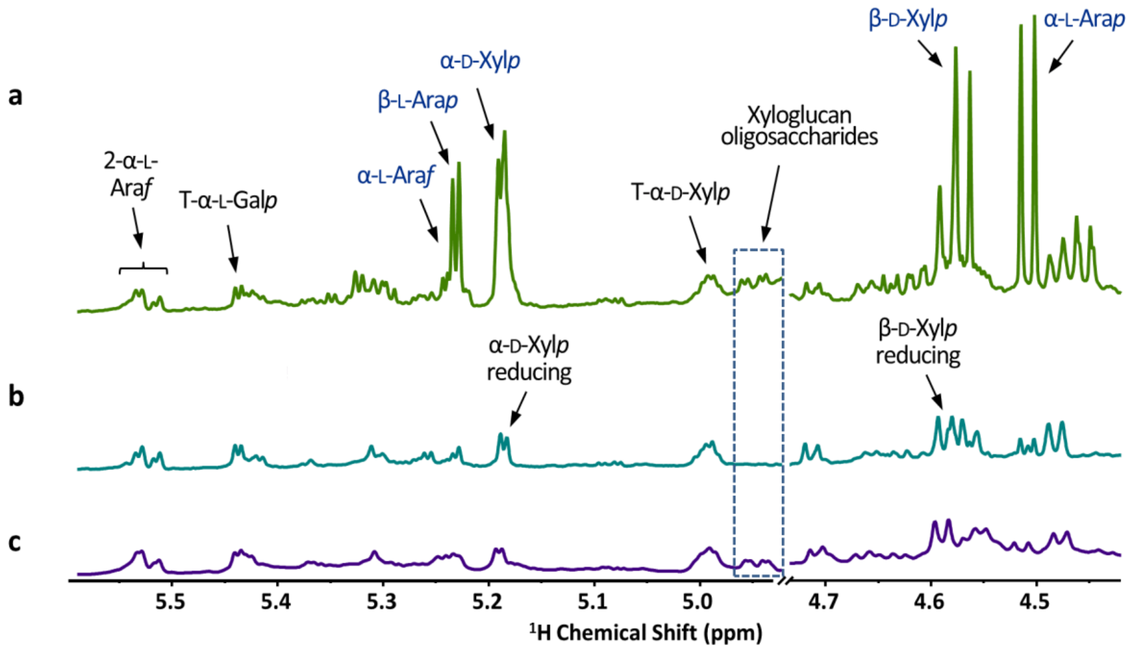

Supplementary Figure 2. NMR analysis of the monoculture and coculture broths. 1D ^{1}H NMR spectra of corn fiber GAX oligosaccharides presented in (a) the Monoculture Broth (b) the *TS*-Coculture Broth and (c) the *TT*-Coculture Broth. The monosaccharides arabinose and xylose (signals labeled in blue) accumulated in the Monoculture Broth but are utilized by *T. saccharolyticum* and *T. thermosaccharolyticum.* Xyloglucan oligosaccharides were identified in the Monoculture Broth. *T. saccharolyticum* utilized the xyloglucan oligosaccharides solubilized by *C. thermocellum. T. thermosaccharolyticum* was unable to utilize them and the oligosaccharides remained in the *TT*-Coculture Broth.

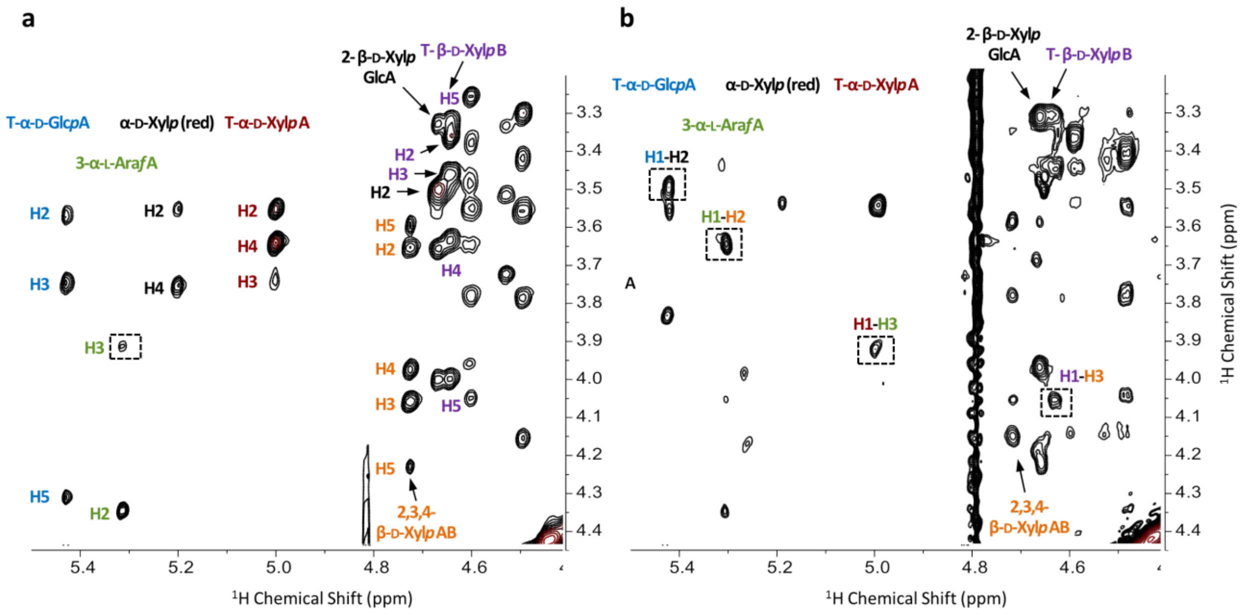

Supplementary Figure 3. Structural characterization of GAX acidic oligosaccharide. This oligosaccharide was isolated from the *TS*-Coculture broth. The labeled cross-peaks in the TOCSY spectrum (a) correspond to the correlations between protons of each glycosyl residue in the oligosaccharide side chains. The signals surrounded by squares in the NOESY spectrum (b) indicated the glycosidic linkages between the residues in these side chains. A chemical structure diagram of the acidic oligosaccharide is showed in the Fig. 3. For the complete list of assignments see the Supplementary Table 2

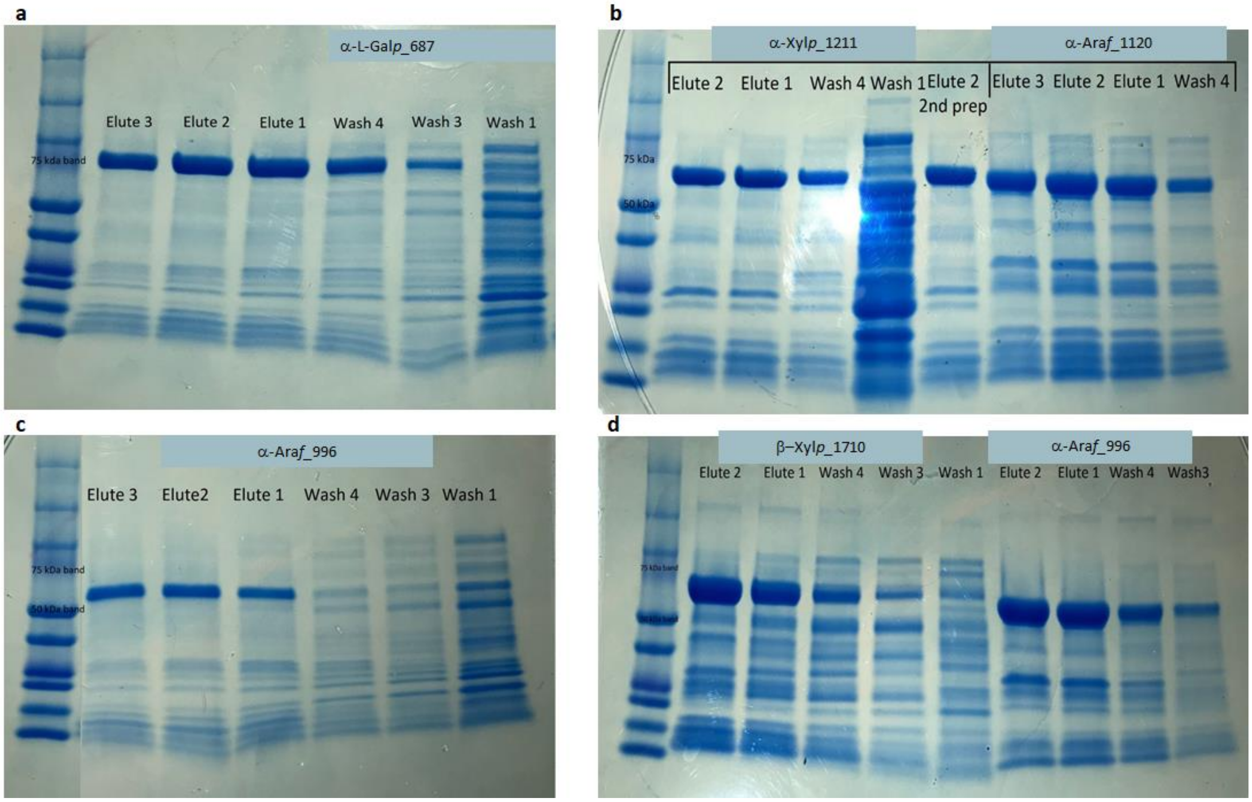

Supplementary Figure 4. Protein gels showing various fractions from the affinity purification of the enzymes. A series of washes and elutions were done in order to get the highest protein purity possible, these were then run on the gel before pooling fractions together based on purity. The size of relevant bands in the protein ladder are given. a) α-L-Gal*p*_687 b) α-Xyl*p*_1211 and α-Ara*f*_1120 c) α-Ara*f*_996 d) β-Xyl*p*_1710 and α-Ara*f*_996

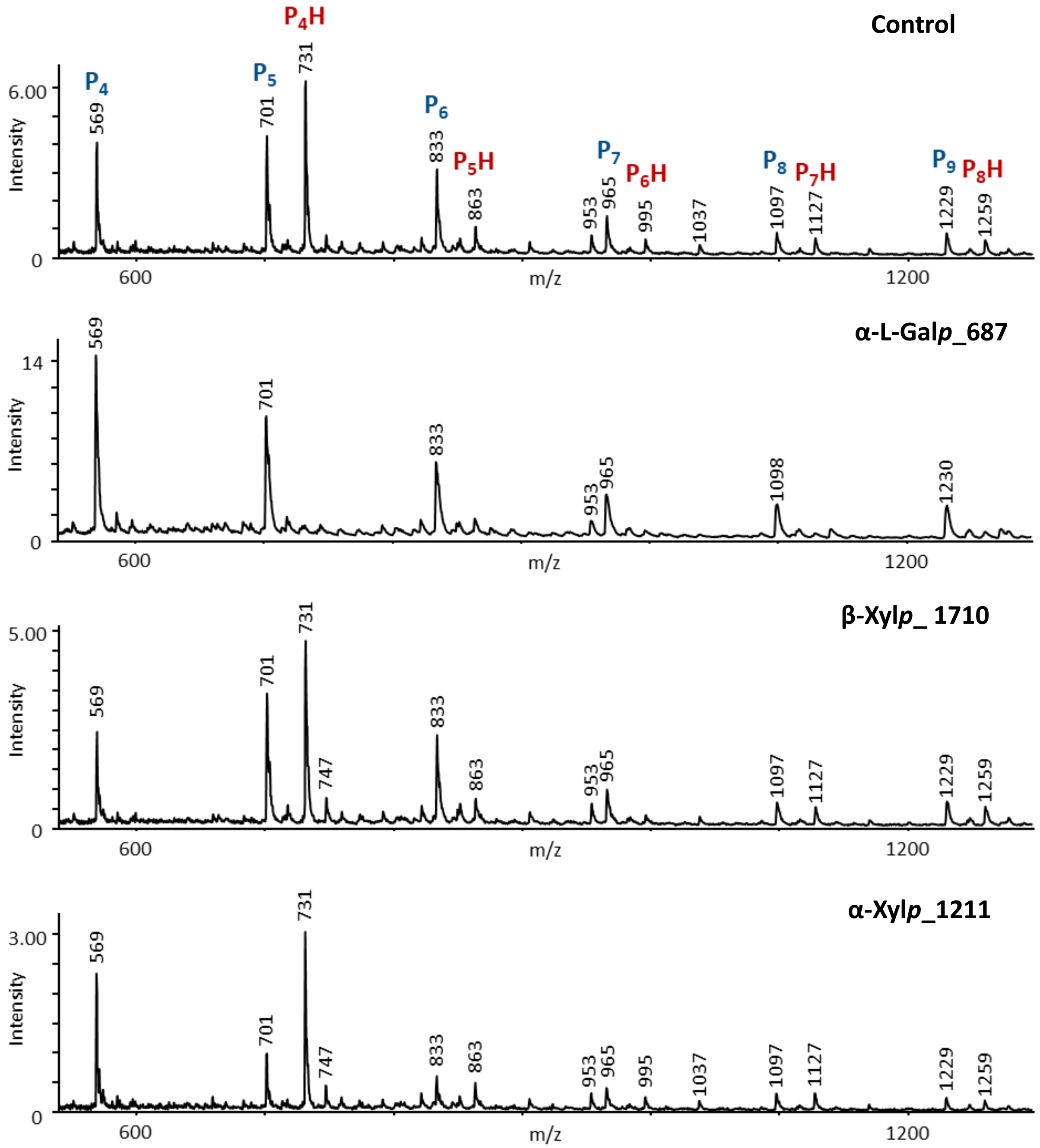

Control
P4 569
P5 701
P4H 731
P6 833
P5H 863
P7 953 965
P6H 995
1037
P8 1097
P7H 1127
P9 1229
P8H 1259
Intensity
m/z
600
1200
α-L-Galp_687
569 701 833 953 965 1098 1230
β-Xylp_ 1710
569 701 731 747 833 863 953 965 1097 1127 1229 1259
α-Xylp_1211
569 701 731 747 833 863 953 965 995 1037 1097 1127 1229 1259

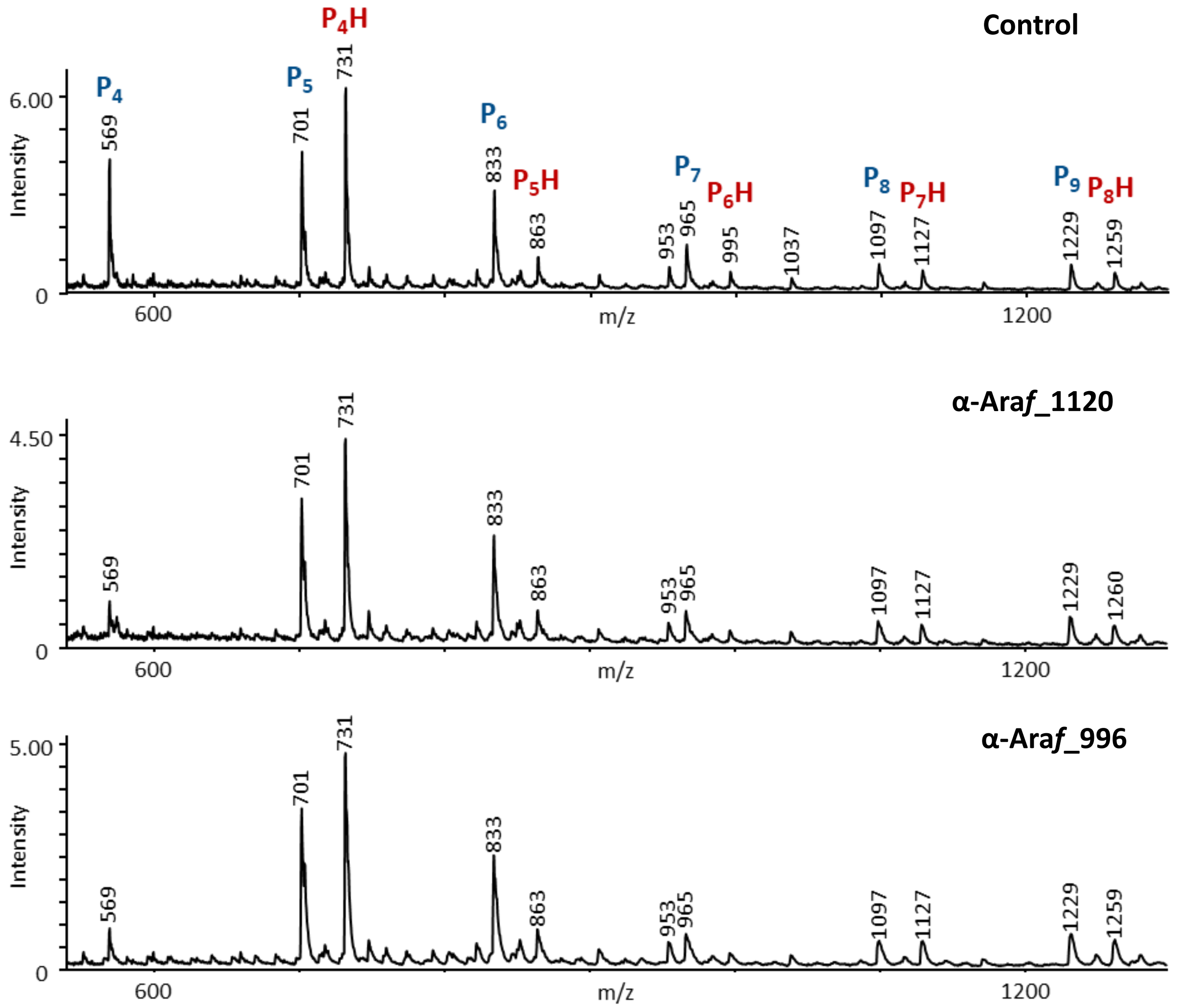

Supplementary Figure 5. Activity of the purified enzymes against neutral oligosaccharides in the *TS.* Coculture Broth. MALDI-TOF mass spectra of the corn fiber GAX neutral oligosaccharides semi-purified from the *TS.* Coculture Broth before and after incubation with each of the purified enzymes for 2 h. P stands for pentose (arabinose or xylose) and H for hexose (galactose).

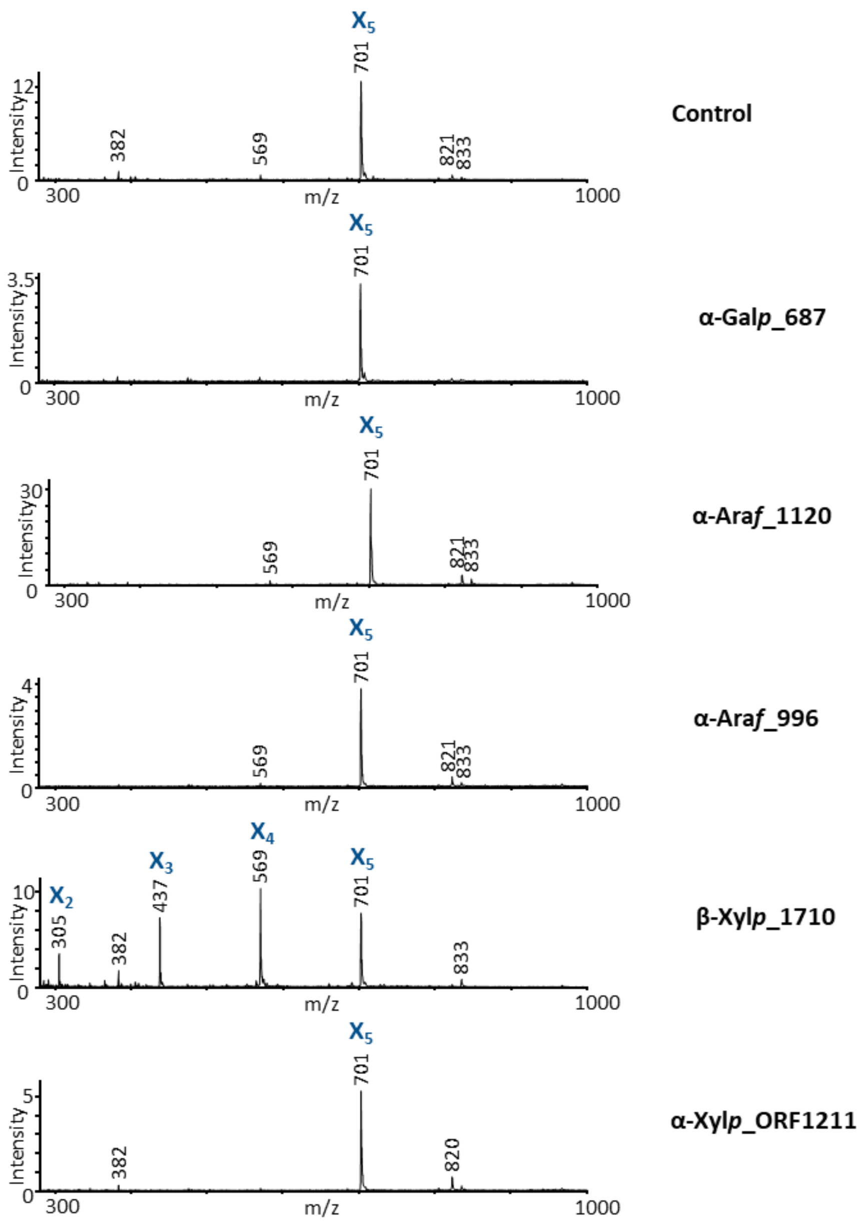

Supplementary Figure 6. Activity of the purified enzymes against linear xylo-oligosaccharides. MALDI-TOF mass spectra of xylopentaose (X_5) incubated for 2 h with each of the purified enzymes. The xylopentaose sample contained traces amounts of X_4 and X_6.

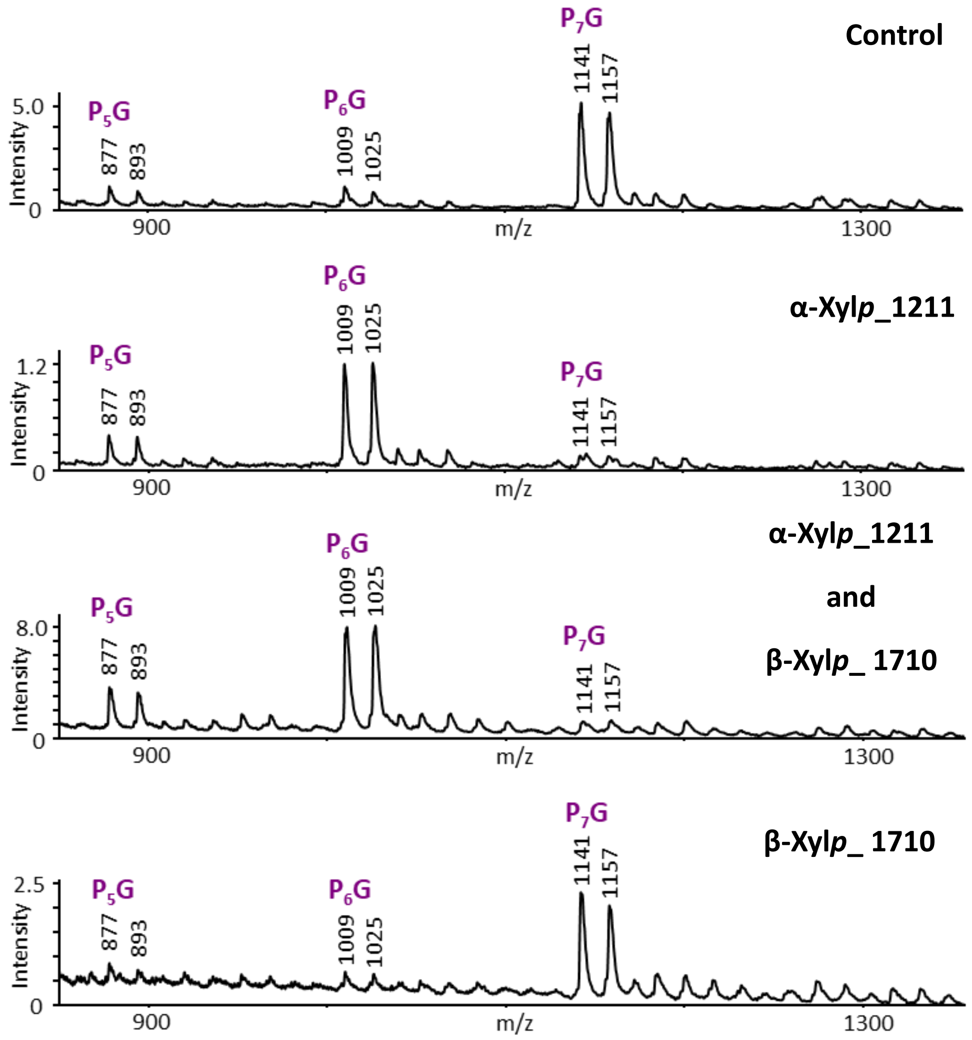

Supplementary Figure 7. Enzymatic hydrolysis of the acidic oligosaccharides. MALDI-TOF mass spectra of the corn fiber GAX acidic oligosaccharides containing the structure *d* after the individual or sequential incubation with α-Xyl*p*_1211 and β-Xyl*p*_1710 for 2 h. The oligosaccharides were isolated from the *TS*-Coculture Broth. P stands for pentose (arabinose or xylose) and G for glucuronic acid.

Supplementary References

1. Sizova, M. V, Izquierdo, J. A., Panikov, N. S. & Lynd, L. R. Cellulose- and xylan-degrading thermophilic anaerobic bacteria from biocompost. *Appl. Environ. Microbiol.* **77**, 2282–91 (2011).

2. Izquierdo, J. A., Pattathil, S., Guseva, A., Hahn, M. G. & Lynd, L. R. Comparative analysis of the ability of *Clostridium clariflavum* strains and *Clostridium thermocellumto* utilize hemicellulose and unpretreated plant material. *Biotechnol. Biofuels* **7**, 136 (2014).

3. Wefers, D. *et al.* Enzymatic mechanism for arabinan degradation and transport in the thermophilic bacterium caldanaerobius polysaccharolyticus *Appl. Environ. Microbiol.* **83**, e00794-17 (2017).
