# Supplementary material for: Development of a thermophilic coculture for corn fiber conversion to ethanol
Source: Nat Commun. 2020 Apr 22;11:1937. doi: 10.1038/s41467-020-15704-z (PMC7176698; doi:10.1038/s41467-020-15704-z)
Supplement: Supplementary file 2 — reporting summary [file 41467_2020_15704_MOESM2_ESM.pdf]

nature research

Corresponding author(s): Lee R. Lynd, Maria J. Pena

Last updated by author(s): Mar 12, 2020

Reporting Summary

Nature Research wishes to improve the reproducibility of the work that we publish. This form provides structure for consistency and transparency in reporting. For further information on Nature Research policies, see Authors & Referees and the Editorial Policy Checklist .

Statistics

For all statistical analyses, confirm that the following items are present in the figure legend, table legend, main text, or Methods section.

n/a	Confirmed	
☐	☒	The exact sample size (*n*) for each experimental group/condition, given as a discrete number and unit of measurement
☐	☒	A statement on whether measurements were taken from distinct samples or whether the same sample was measured repeatedly
☒	☐	The statistical test(s) used AND whether they are one- or two-sided<br>*Only common tests should be described solely by name; describe more complex techniques in the Methods section.*
☒	☐	A description of all covariates tested
☐	☒	A description of any assumptions or corrections, such as tests of normality and adjustment for multiple comparisons
☐	☒	A full description of the statistical parameters including central tendency (e.g. means) or other basic estimates (e.g. regression coefficient) AND variation (e.g. standard deviation) or associated estimates of uncertainty (e.g. confidence intervals)
☒	☐	For null hypothesis testing, the test statistic (e.g. *F*, *t*, *r*) with confidence intervals, effect sizes, degrees of freedom and *P* value noted<br>*Give P values as exact values whenever suitable.*
☒	☐	For Bayesian analysis, information on the choice of priors and Markov chain Monte Carlo settings
☒	☐	For hierarchical and complex designs, identification of the appropriate level for tests and full reporting of outcomes
☒	☐	Estimates of effect sizes (e.g. Cohen's *d*, Pearson's *r*), indicating how they were calculated

Our web collection on statistics for biologists contains articles on many of the points above.

Software and code

Policy information about availability of computer code

Data collection	-NMR data collection software: VnmrJ version 4.2 Revision A (Agilent Technologies, Inc., https://www.agilent.com)<br>-MALDI TOF-MS data collection software: Bruker Daltonik FlexControl version 3.0 (Bruker Corporation, https://www.bruker.com)<br>-Empower 2 software for instrument control and data analysis (Waters Corporation, Milford, MA, USA)
Data analysis	-NMR data analysis software: MestReNova version 12.0.3-21384 (Mestrelab Research S.L., Spain, http://mestrelab.com/software/mnova)<br>-Empower 2 software for instrument control and data analysis (Waters Corporation, Milford, MA, USA)

For manuscripts utilizing custom algorithms or software that are central to the research but not yet described in published literature, software must be made available to editors/reviewers. We strongly encourage code deposition in a community repository (e.g. GitHub). See the Nature Research guidelines for submitting code & software for further information.

Data

Policy information about availability of data

All manuscripts must include a data availability statement. This statement should provide the following information, where applicable:

- Accession codes, unique identifiers, or web links for publicly available datasets
- A list of figures that have associated raw data
- A description of any restrictions on data availability

Data supporting the findings of this work are available within the paper and its Supplementary Information files. The source data underlying Table 1, 2, 4 and 5 as well as Fig. 5 are provided as a Source Data file. The genomic DNA for strains LL1354 and LL1355 was sequenced by the Joint Genome Institute (Bioprojects PRJNA371193 [https://www.ncbi.nlm.nih.gov/bioproject/371193] and PRJNA345749 [https://www.ncbi.nlm.nih.gov/assembly/GCA_003945225.1]; nucleotide accession number NZ_RSDX01000001 [https://www.ncbi.nlm.nih.gov/nuccore/NZ_RSDX01000001])Bioprojects PRJNA371193 and PRJNA345749; nucleotide accession number NZ_RSDX01000001). The plasmids used to express LL1355 enzymes in T. thermosaccharolyticum were numbered pLL1270-pLL1277 and have been deposited with Addgene with the IDs 129281, 129282, 129287, 129288, 129385, 129386, 129407 and, 129408.

Field-specific reporting

Please select the one below that is the best fit for your research. If you are not sure, read the appropriate sections before making your selection.

- [x] Life sciences
- [ ] Behavioural & social sciences
- [ ] Ecological, evolutionary & environmental sciences

For a reference copy of the document with all sections, see nature.com/documents/nr-reporting-summary-flat.pdf

Life sciences study design

All studies must disclose on these points even when the disclosure is negative.

Sample size	A variety of different measurements were made in this study, and sample sizes were small, from 1-3
Data exclusions	No data were excluded from the analyses.
Replication	Experiments were conducted on at least two different days and data is reported by averaging values obtained with the exception of Supplementary Figure 5 where a qualitative inference sufficed. Number of replicates were chosen so as to be enough to prove significance.
Randomization	We did not use experimental groups in our study
Blinding	Not applicable as experimental groups were not used.

Reporting for specific materials, systems and methods

We require information from authors about some types of materials, experimental systems and methods used in many studies. Here, indicate whether each material, system or method listed is relevant to your study. If you are not sure if a list item applies to your research, read the appropriate section before selecting a response.

Materials & experimental systems

n/a	Involved in the study	
x		Antibodies
x		Eukaryotic cell lines
x		Palaeontology
x		Animals and other organisms
x		Human research participants
x		Clinical data

Methods

n/a	Involved in the study	
x		ChIP-seq
x		Flow cytometry
x		MRI-based neuroimaging
